# Multiple Sources of Introduction of North American *Arabidopsis thaliana* from across Eurasia

**DOI:** 10.1093/molbev/msab268

**Published:** 2021-09-09

**Authors:** Gautam Shirsekar, Jane Devos, Sergio M Latorre, Andreas Blaha, Maique Queiroz Dias, Alba González Hernando, Derek S Lundberg, Hernán A Burbano, Charles B Fenster, Detlef Weigel

**Affiliations:** 1 Max Planck Institute for Developmental Biology, Tübingen, Germany; 2 Centre for Life’s Origin and Evolution, University College London, London, United Kingdom; 3 Oak Lake Field Station, Department of Natural Resource Management, South Dakota State University, Brookings, SD, USA

**Keywords:** *Arabidopsis thaliana*, population genetics, admixture, nonnative species, migration

## Abstract

Large-scale movement of organisms across their habitable range, or migration, is an important evolutionary process that can shape genetic diversity and influence the adaptive spread of alleles. Although human migrations have been studied in great detail with modern and ancient genomes, recent anthropogenic influence on reducing the biogeographical constraints on the migration of nonnative species has presented opportunities in several study systems to ask the questions about how repeated introductions shape genetic diversity in the introduced range. We present an extensive overview of population structure of North American *Arabidopsis thaliana* by studying a set of 500 whole-genome sequenced and over 2,800 RAD-seq genotyped individuals in the context of global diversity represented by Afro-Eurasian genomes. We use methods based on haplotype and rare-allele sharing as well as phylogenetic modeling to identify likely sources of introductions of extant N. American *A. thaliana* from the native range in Africa and Eurasia. We find evidence of admixture among the introduced lineages having increased haplotype diversity and reduced mutational load. We also detect signals of selection in immune-system-related genes that may impart qualitative disease resistance to pathogens of bacterial and oomycete origin. We conclude that multiple introductions to a nonnative range can rapidly enhance the adaptive potential of a colonizing species by increasing haplotypic diversity through admixture. Our results lay the foundation for further investigations into the functional significance of admixture.

## Introduction

*Arabidopsis thaliana* is predominantly a human commensal that is native to Africa and Eurasia. Its demographic history is filled with episodes of range expansions, bottlenecks, migrations, and admixture. Current models of *A. thaliana*’s population history highlight the recurrent theme of lineage migration and admixture with locally adapted genotypes in the native range of the species (Durvasula et al. 2017; Lee et al. 2017; Zou et al. 2017; Fulgione and Hancock 2018; Fulgione et al. 2018; Hsu et al. 2019). A further opportunity to learn about the impact of demographic processes and selection in *A. thaliana* arises from its relatively recent colonization of North America.

When a species is introduced outside its native range, where its long-term eco-evolutionary history has been established, different factors determine how well the introduced population adapts to the new environment. These factors include, but are not limited to history of introduction, founder effects, and strength of natural selection (Colautti and Lau 2015; Estoup et al. 2016). In the post-Columbian era, *A. thaliana* has benefited from mostly unidirectional cross-continental species movement facilitated by human migrations to N. America (La Sorte et al. 2007; Winter et al. 2010). Thus, the N. American *A. thaliana* metapopulation presents a unique natural experiment for studying the role of history in explaining extant diversity and understanding how the colonizers have thrived despite population bottlenecks and seemingly low genetic diversity, also known as genetic paradox of invasion (Allendorf and Lundquist 2003). Important questions that can be addressed using this study system are: How much of the native diversity was introduced to N. America? How much new diversity has been generated in situ through mixing of lineages that originated from distant parts in the native range? How much of the observed diversity is due to selection?

*Arabidopsis thaliana* has become established across much of N. America. Coarse-scale population structure analysis of N. American individuals with 149 single-nucleotide polymorphism (SNP) markers has revealed the presence of a dominant lineage “Haplogroup1” (Hpg1) (Platt et al. 2010). Patterns of mutation accumulation in the genomes of pure Hpg1 individuals have supported an arrival in N. America about 400 years ago, soon after Europeans started to arrive en masse on the continent. A parsimonious explanation of the ubiquitous nature of this lineage could be that it was the earliest to be introduced to N. America (Exposito-Alonso, Becker, et al. 2018). So far, little consideration has been given to the supposedly subsequent arrival of other lineages, their origins in the native range, their fate as migrations continued during the past centuries, and how the genomes of the current N. American population have been shaped by processes such as admixture and adaptation.

We present the fine-scale population structure of the N. American *A. thaliana* population as viewed through the lens of range-wide genetic diversity of the species. Using genomes of *A. thaliana* individuals collected from the Midwest, the Eastern Seaboard, and the North–East of the current United States, we infer possible sources of ancestry based on haplotype-sharing, phylogenetic tree-based modeling, and rare allele sharing (RAS) with the worldwide data set. We also describe how admixture in this predominantly selfing species is generating new haplotype diversity and how admixture affects the fate of deleterious mutations and allows selection on immunity-related loci. The work presented here shows that increased global connectivity through the past two centuries has made species invasions from across the species range common and could have accelerated invasion of N. American habitat by avoiding the genetic paradox of invasion. Further, our work highlights that processes such as admixture and selection that determine success of lineages in the native range play a similarly important role in the nonnative range.

## Results

### An Overview of Population Structure and Genetic Variation from RAD-Seq

**Fig. 1.**
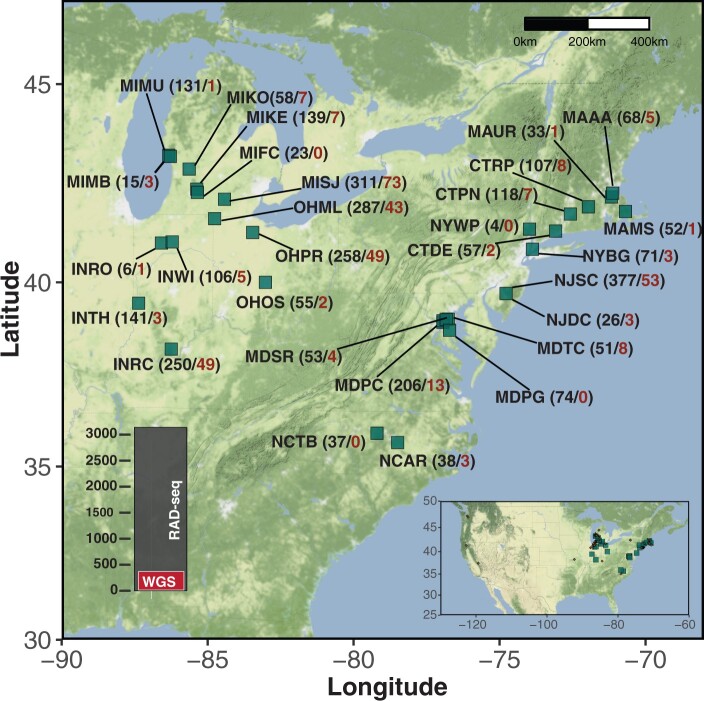
Locations and number of sampled individuals. Abbreviations of the locations sampled are shown along with the number of RAD-sequenced samples (in black) and the number of whole-genome sequenced (WGS) samples (in red). Left inset: bar plot of total number of samples sequenced. Right inset: sampling area in the context of N. America.

We collected *A. thaliana* samples across an area of about 1,200 by 900 km in the Eastern United States during the spring seasons (mid-March to early June) of 2014, 2015, and 2016 (fig. 1 and supplementary table S1*a* and *b*, Supplementary Material online). We genotyped these samples using a RAD-seq implementation of reduced representation sequencing (Miller et al. 2007). After filtering for sequencing output and quality, we retained 2,861 individuals, which shared 4,907 polymorphic SNPs. In order to compare the population structure and genetic diversity in our N. American to the global Afro-Eurasian collection (AEA), we used data from the 1001 Genomes project (1001 Genomes Consortium 2016) in addition to whole-genome sequences (WGS) from 13 Irish (this work), ten African (Durvasula et al. 2017), and five Yangtze River basin accessions (Zou et al. 2017). From these AEA individuals, information on the 4,907 polymorphic SNPs found in our N. American individuals (average depth ∼36×) were extracted and merged with the N. American data set for further analysis. Although pairwise similarity using “identity-by-state” (IBS) and “identity-by-descent” (IBD) across the genome is greater in N. American than in AEA individuals, genetic variation relative to AEA individuals could nevertheless be observed in N. American individuals with principal component analysis (PCA) (supplementary fig. S1*A*, Supplementary Material online). North American individuals in our collection were genetically much more diverse than the N. American individuals previously sequenced as part of the 1001 Genomes Project (supplementary fig. S1*B*, Supplementary Material online).

### Diversity of N. American Haplogroups

We first used RAD-seq to rapidly genotype thousands of individuals, but because of its inherent biases (low density of markers, strand-bias, underestimation of genetic diversity), these data are not well suited for fine-scale, quantitative population genomic analyses (Arnold et al. 2013; Cariou et al. 2016; Lowry et al. 2017). We therefore selected and sequenced a subset of distantly related individuals with WGS approach, at an average of ∼8× coverage (0.63–43.91*x*, median=5.81*x*). A PCA of 500 N. American individuals, including a subset of previously analyzed herbarium individuals (Exposito-Alonso, Becker, et al. 2018) (supplementary fig. S1*C*, Supplementary Material online), resulted in an arrangement in which most individuals were found along distinct clines. We decided to explore this population structure in detail using different complementary population genetic methods.

**Fig. 2.**
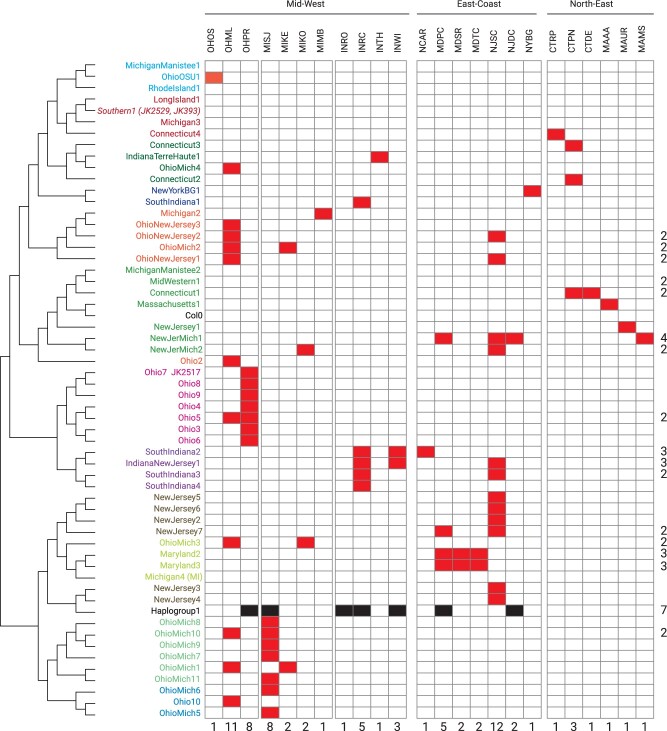
Identification of North American *groups* based on haplotype sharing and their distribution in different populations. Collapsed fineSTRUCTURE tree generated by merging North American individuals into groups (herbarium individuals are denoted by JKxxx) based on their coancestry (derived using CHROMOPAINTERv2, coancestry matrix in supplementary fig. S3, Supplementary Material online). Last row of numbers represents the total count of *groups* present in the population, and last column of numbers represents the number of populations in which a specific *group* is present (here a *group* present in a single population is not counted, count=1).

Finer-scale population structure can be revealed by explicitly modeling the effects of linkage disequilibrium (LD) and clustering individuals based on their shared ancestry that emerges after accounting for LD (Busby et al. 2015; Leslie et al. 2015; Montinaro et al. 2015). Therefore, we hierarchically partitioned the N. American individuals into 58 clusters (from here on called *groups*) using a coancestry matrix derived using CHROMOPAINTER v2 and MCMC-based clustering in fineSTRUCTURE (Lawson et al. 2012) (supplementary fig. S3, Supplementary Material online). Haplogroup1 (Hpg1) is the most frequently observed *group* across the sampled populations (fig. 2), consistent with previous observations (Platt et al. 2010; Exposito-Alonso, Becker, et al. 2018). OHML (Ohio) and NJSC (New Jersey) had the highest within-population haplotype diversity, with 11 and 12 *groups*. Several *groups*, such as OhioNewJersey2, IndianaNewJersey1, and NewJerMich1, were found in populations from geographically distinct regions (fig. 2).

We further analyzed the genetic relationships among these *groups* using several complementary approaches. Treemix (Pickrell and Pritchard 2012), without considering migration edges, reconstructed relationships among the *groups* (supplementary fig. S4*A*, Supplementary Material online), similar to the topology inferred by fineSTRUCTURE clustering (supplementary fig. S3*B*, Supplementary Material online). Notably, residuals from the fitted model with high positive values indicated that the fit could be improved by including admixture edges among the *groups* (supplementary fig. S4*B*, Supplementary Material online). High positive residual values between Hpg1, which is the omni-present *group* with high frequency and other *groups*, suggested possible gene flow between them.

Stochastic changes in allele frequency, as a result of the neutral process of drift, hold information about shared ancestry. We therefore estimated values for the *f*_3_*-*outgroup statistic (Raghavan et al. 2014) to understand the shared drift among *groups* relative to an outgroup (individuals of relict ancestry). Relicts comprise highly diverged individuals from ice age refugia (Lee et al. 2017) and therefore were chosen as an outgroup. Indeed, some of the N. American groups (OhioMich1, SouthIndiana4, and Ohio7) along with Hpg1 shared excess drift with other *groups* (supplementary fig. S5, Supplementary Material online). As the *f*_3_*-*outgroup test identifies the closest relative population and does not itself point to the admixture, we applied *f*_3_ statistic to explore the possibility of admixture among these groups (Patterson et al. 2012). We calculated values for the *f*_3_ statistic in all trios (*groupA*, *groupB*: *groupTest*) of N. American *groups* to detect whether *groupTest* was admixed between *groupA* and *groupB*. There were several *groupTest* examples with negative f3 scores and *Z* scores below −3 in several trios (supplementary fig. S6*A* and table S2, Supplementary Material online). In several cases, Hpg1 emerged as a putative source (as either *groupA* or *groupB*) (supplementary table S2, Supplementary Material online). To investigate this in more detail, we calculated the shared drift of Hpg1 relative to the other N. American *groups*. We found more *groups* with a gradient of shared drift with Hpg1; Massachusetts1 was one of the *groups* with least shared drift (supplementary fig. S6*B*, Supplementary Material online). Therefore, we calculated the ABBA-BABA statistic (*D*-statistic) in the form of (*Massachusetts1, Test: Haplogroup1, Relicts*) to learn the extent of gene flow between Hpg1 and other N. American *groups* (supplementary fig. S6*C*, Supplementary Material online). Many *groups* showed significantly more ABBA sites (*Z* score < −3) than BABA sites, confirming the contribution of Hpg1 ancestry to the genetic makeup of these *groups*.

### Contribution of Distinct Sources of Ancestry to N. American Diversity

North American groups vary in terms of their drift relative to the earliest arrival, Hpg1, which suggests that there have been multiple introductions of *A. thaliana* to N. America. It is also unclear whether the observed haplogroups already existed in Eurasia, or whether they only formed by intercrossing in N. America. We therefore wanted to learn whether N. American extant haplogroups include ancestry from different geographic regions in Eurasia. We first excluded lineages that showed evidence of recent admixture (*groups*

with significantly negative *f*_3_*-*scores), and we then applied statistical procedures based on shared haplotype chunks (fineSTRUCTURE), shared drift (*f*_3_*-*outgroup, *D*-statistic, and qpWave) and enrichment of rare alleles with respect to the AEA haplotype diversity to identify sources of ancestry in Eurasia based on WGS from AEA individuals (*n* = 928) (1001 Genomes Consortium 2016). We traversed the genomes of N. American individuals to assign local ancestry along each chromosome. To this end, we performed haplotype-based inference in three steps: 1) Paint each AEA individual against the others (excluding itself) with CHROMOPAINTER v2, 2) Based on haplotype sharing, cluster individuals into *subclusters* using fineSTRUCTURE. These *subclusters* were then grouped into *clusters*, and *clusters* were further grouped into *regions* (supplementary fig. S7, Supplementary Material online; details of these hierarchical partitions for each AEA individual are given in supplementary table S3, Supplementary Material online). 3) We chose 15 representative individuals per AEA *region* and estimated an ancestry profile for individual N. American recipients.

**Fig. 3.**
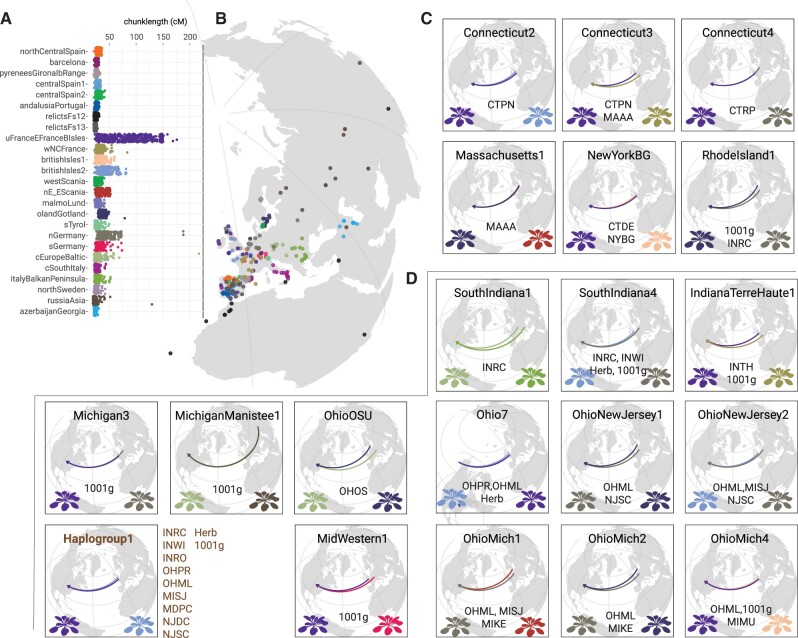
Chromosome painting of N. American *groups* with Afro-Eurasian (AEA) *regions* as donors. (*A*) Copying profile of the N. American individuals inferred with CHROMOPAINTERv2 using a reference panel of individual haplotypes belonging to different AEA *regions*, each dot represents an individual (cumulative genomic segment length copied is in centiMorgans). (*B*) Geographic locations of the AEA individuals used in the reference panel (colored by *region*). (*C, D*) Two major contributions from AEA *regions* to N. American *groups* found on Eastern Seaboard-Northeast and Midwest. Arrows point from the mean geographic position of the AEA *regions* to that of the N. American *groups* (colors of the contributing *region* are the same as in panel [*A*]. Credit for original design of *A. thaliana* rosettes: Frédéric Bouché).

Figure 3*A* shows these inferred ancestry profiles for the N. American individuals. It can be seen that although the majority of groups are enriched for Upper/EastFranceBritishIsles ancestry, other British Isles *regions* (BritishIsles1 and BritishIsles2) also feature significantly across several groups (fig. 3*C and D*; supplementary fig. S8, Supplementary Material online). Apart from these, some N. American *groups* such as MichiganManistee1, OhioOSU, and SouthIndiana1 had substantially higher contributions from East European *regions* such as RussiaAsia, CentralEurope/Baltic, and Italy/BalkanPeninsula. NorthGermany and SouthGermany *regions* have contributed to the ancestry of OhioMich1, RhodeIsland1, and Mid-Western1 *groups* (fig. 3*C and D*; supplementary fig. S8, Supplementary Material online). These results also highlight how geo-genetically distant AEA ancestries could be found within the same population (INRC) or within the same regions (Midwest) in N. America.

We explored these haplotype-sharing patterns further by measuring shared drift between a test N. American *group* and 158 *subclusters* of AEA individuals using *f*_3_-outgroup statistic of the form *test, subcluster*; relictsFs12-3 (We chose relictsFs12-3 as an outgroup as it is a highly diverged *subcluster* comprising relict population individuals) and building a maximum likelihood (ML) tree by fitting Treemix (Pickrell and Pritchard 2012) model without any admixture edges. At a coarser scale, the results agree with the haplotype-based inferences. Shared allelic drift measured with *f*_3_-outgroup statistic and captured in the ML tree showed that the current N. American *groups* are related to the AEA *subclusters* that belonged to either western, central, or eastern Europe (supplementary figs. S9 and S10, Supplementary Material online). We also observed these patterns of relatedness qualitatively in a PCA plot where we projected N. American individuals into PC space occupied by AEA individuals (supplementary fig. S11*A*, Supplementary Material online). Even finer details became apparent with uniform manifold approximation and projection (UMAP) embeddings (McInnes et al. 2018) (supplementary fig. S11*B*, Supplementary Material online) derived from the first 50 PC components of all the individuals (without projection).

**Fig. 4.**
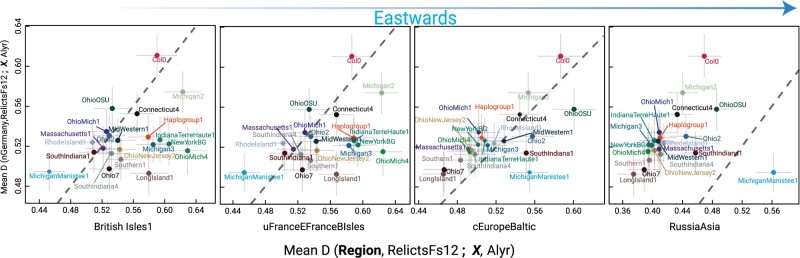
Multiple sources of origin of N. American haplogroups. Biplot of mean *D*-statistics of N. American haplogroups (X) with *subclusters* comprising NorthGermany region (*NorthGermany*, *RelictsFs12*; *X*, *A.lyrata*), against mean *D*-statistics of *subclusters* comprising different *regions* in an eastward direction (*testRegion*, RelictsFs12; *X*, *A.lyrata*). Vertical and horizontal bars represent the spread of *D*-statistics from member *subclusters* of each *region*.

The coarse patterns of shared ancestry emerging from *f*_3_*-*outgroup statistic, PCA projection, and UMAP embeddings were tested in a more systematic way by evaluating the “treeness” of different topological configurations. We first calculated *D*-statistic (Green et al. 2010) for all the N. American nonadmixed *groups* (X) in the form of NorthGermany, RelictsFs12; X, *A. lyrata* (*Arabidopsis lyrata* as a closest relative to *A. thaliana*; Schmickl et al. 2010; was chosen as an outgroup). NorthGermany *region* was chosen because of its central geographic location among the possible sources of N. American *A. thaliana*. We then calculated *D-*statistics for the N. American *groups* by replacing NorthGermany with four *regions*: BritishIsles1, Upper/EastFranceBritishIsles, CentralEurope/Baltic, and RussiaAsia. We then plotted *D*-statistics with replacement *regions* to *D*-statistics obtained using NorthGermany separately to understand region specific drift (Ebenesersdóttir et al. 2018). These biplots (fig. 4*C*) clearly differentiate western and eastern European sources of ancestry in N. American *A. thaliana*. OhioOSU, Ohio2, SouthIndiana1, and MichiganManistee1 clearly showed the relative eastern European ancestry component. The analysis also revealed that Col-0, the reference genome accession for *A. thaliana* research, shares significant ancestry with individuals from NorthGermany, confirming the origin of Col-0 in or near Germany (Rédei 1992). Despite constrained “tree” topologies explored, since we used *subclusters* to estimate the *D*-statistic, it allowed us to capture variation in the shared drift (horizontal and vertical bars in fig. 4) experienced by a target N. American *group* with a given AEA *region.*

We extended this analysis using qpWave (Reich et al. 2012) to test whether any two N. American *groups* would be symmetrically related to a set of outgroups (AEA *regions*). Specifically, we tested whether a set of *f*_4_-statistics comprising two N. American *groups* across a set of layer1 outgroups (AzerbaijanGeorgia, Barcelona, NorthSweden, NorthWestEngland, Relicts Fs13, SouthTyrol, WestScania, and West/NorthCentralFrance) makes a matrix of rank 0 (same wave of ancestry) (supplementary table S4, Supplementary Material online). We then tested whether addition of an extra outgroup region (consisting of putative sources of ancestry) to the layer 1 outgroup set affected the symmetry of shared ancestry. If the two test N. American *groups* are differentially related to the extra outgroup region, then it would increase the rank of the original matrix of *f*_4_-statistics (rejection of rank 0), indicating distinct streams of the ancestry among the test *groups*. We added an extra outgroup from additional regions of BritishIsles2, ItalyBalkanPeninsula, NorthGermany, RussiaAsia, and Upper/EastFranceBritishIsles one-by-one. Adding these putative source regions affected the symmetrical relationships observed with our original outgroup set. Except in the case of SouthIndiana4 and Ohio7, all the N. American *group* combinations showed asymmetric relationships (rejection of rank 0) with these extra outgroups (supplementary table S4, Supplementary Material online). These results validated the findings from qualitative observations made with PCA projection, and UMAP embeddings. It further confirmed results obtained from *f*_3_-outgroup statistic, ML tree, and *D-*statistics analysis, that the N. American *A. thaliana groups* have ancestral components from western Europe (mainly British Isles), central Europe, and eastern Europe.

More subtle patterns of ancestry can be inferred by finding rare variants (Schiffels et al. 2016) from AEA that have risen to higher frequency in N. American individuals. Because we had moderate- to high-coverage whole genomes of the AEA and N. American individuals, we could use such rare variants to independently ascertain the results obtained from the haplotype-based ancestry inference and shared ancestry-based inference, mostly on moderate to high frequency alleles. We identified variants from AEA individuals with frequency of 1% or lower and tracked their enrichment in the N. American *groups*. We found that N. American *groups* have accumulated rare alleles from different AEA *subclusters* (supplementary fig. S12, Supplementary Material online). Whereas several N. American *groups* have inherited rare alleles from British Isles *subclusters*, *groups* RhodeIsland1, MichiganManistee1, OhioOSU, SouthIndiana1, and OhioMich1 have accumulated rare alleles from central/eastern European subclusters, whereas Hpg1 has accumulated a significant number of rare alleles from *subclusters* from the Upper/EastFrance/BritishIsles *region*. Taken together, this analysis confirmed that N. America was colonized by *A. thaliana* in multiple waves with distinct sources of ancestry.

### Environmental Conditions at Source and Success of Colonizing Lineages

As we had inferred the shared ancestry of the colonizing lineages with different complementary methods, we hypothesized that besides human-assisted migration, environmental similarity between putative source *subclusters* and colonizing lineages contributed to successful colonization of the lineages. To test this hypothesis, we fit a regression model to predict shared ancestry with AEA *subclusters* (as measured by *f*_3_-outgroup statistics of the form test *N. American group, AEA subcluster: RelictsFs12_3 (outgroup)*, value of the statistic is proportional to the shared ancestry between the populations relative to the outgroup), using linear combinations of four environmental variables: average temperature (tavg), precipitation (prec), solar radiation (srad), and water vapor pressure (vapr). *Arabidopsis thaliana* shows significant local adaptation to climate (Exposito-Alonso, Vasseur, et al. 2018; Fulgione and Hancock 2018), thus the choice of these four variables should provide a general climatic niche. We used Bayesian multilevel modeling (bMLM) framework (Gelman 2006) to understand each N. American group’s environmental association with its putative source AEA *subclusters* without ignoring the environmental association to the entire cohort of N. American *groups*.

Population-scale coefficients for the environmental variables precipitation (mm) and water vapor pressure (kPa) revealed that environmental dissimilarity calculated by Euclidean distance between each N. American *group* and AEA *subcluster* is negatively correlated with the *f*_3_-outgroup statistics (table 1). Although average temperature dissimilarity is slightly negatively correlated with *f*_3_-outgroup statistics, the compatibility interval with the model is large, with slightly positive correlation in posterior distribution. Upon closer examination of the coefficients estimated for individual N. American *groups*, it can be seen that precipitation and water vapor pressure dissimilarity is negatively correlated with the *f*_3_-outgroup statistic for all *groups* but MichiganManistee1 (supplementary fig. S13, Supplementary Material online). Overall the general trend of negative correlation of the linear combination of the dissimilarity of the variables (average temperature, precipitation, solar radiation, and vapor pressure) to the *f*_3_-outgroup statistic can be captured with the individual estimates sampled from the posterior distribution (supplementary fig. S14, Supplementary Material online).

**Table 1.** Posterior Summary of the Regression Coefficients for Environmental Variables.

Parameter	Mean	SD	hdi_3%	hdi_97%	*^R*
$\bar{a}$	0.1390	0.0920	−0.0280	0.3190	1.0000
${\bar{\beta }}_{\text{Tavg}}$	−0.0640	0.0960	−0.2420	0.1170	1.0000
${\bar{\beta }}_{\text{Prec}}$	−0.1800	0.0910	−0.3530	−0.0080	1.0000
${\bar{\beta }}_{\text{Srad}}$	−0.0010	0.0910	−0.1800	−0.1600	1.0000
${\bar{\beta }}_{\text{Vapr}}$	−0.2320	0.0960	−0.4080	−0.0490	1.0000
σ	0.3950	0.0110	0.3730	0.4160	1.0000

Note.—Bayesian multilevel model-based pooled estimates of regression coefficients for environmental variables *T*_avg_ (°C), precipitation (mm), solar radiation (kJ^ ^m^−2 ^day^−1^), and water vapor pressure (kPa). *f*_3_*-*outgroup statistic of each *N*. American group to every AEA subcluster (outgroup: relicts Fs12_3) was used as a Student’s *t*-distributed dependent variable.

The negative correlation between environmental dissimilarity and shared ancestry led us to hypothesize that in reduced dimensional space of environmental variables (average temperature, precipitation, and vapor pressure), N. American *groups* should be closer to their source AEA *subclusters*. To test this, we performed UMAP on the standardized values for environmental variables for N. American *groups* and AEA *clusters* together, followed by hierarchical clustering on the reduced environmental space (see details in the Supplementary Material online). We observed that the N. American groups and their putative source *clusters*, as inferred by population genomic approaches (specifically *subclusters* from Upper/EastFranceBritishIsles, NorthGermany, SouthGermany, BritishIsles1, BritishIsles2, and CentralEurope/Baltic *regions*) occupied similar space in the UMAP embeddings (supplementary fig. S16, Supplementary Material online) and were in the same major clades (supplementary fig. S17, Supplementary Material online), thus confirming that overall environmental similarity between source populations and N. America might be an important contributor to the success of colonization.

### Effect of Admixture on Deleterious Mutations

**Fig. 5.**
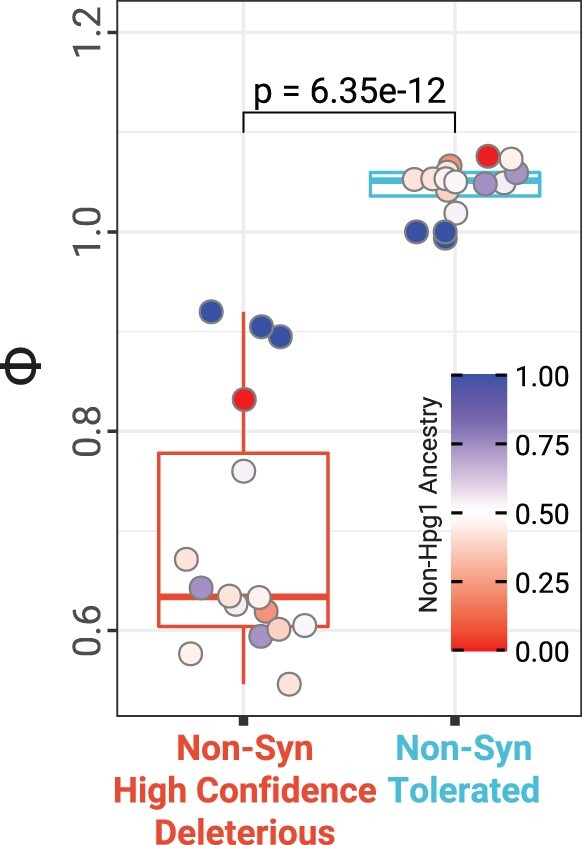
Scaled fixed-to-total derived alleles ratios (Φ) for N. American *groups* across nonsynonymous mutation categories. Scaled fixed-to-total derived alleles ratio (Φ) for each *group* in populations OHPR, INRC, MISJ, and NJSC for nonsynonymous mutation categories was calculated, scaled by the fixed-to-total derived alleles ratio for synonymous mutations. Points represent different *groups* in the focal populations and colors of the individual points represent its non-Hpg1 ancestry proportion, as indicated on the scale at the right. Fixed derived alleles are alleles with frequency=1. *P* value is from Welch’s *t*-test.

Evolutionary theory predicts that during range expansions and new colonizations deleterious mutations accumulate gradually and steadily, resulting in increased mutational load that can be reduced again by outcrossing (Peischl et al. 2013). We hypothesized that the levels of mutational load in N. American individuals would be related to rates of historic outcrossing as inferred from admixture. To test this, we chose individuals from populations INRC, MISJ, NJSC, and OHPR because of: 1) Presence of earliest colonizing lineage Hpg1 and 2) two-way admixture events between Hpg1 and non-Hpg1 *groups* (supplementary fig. S6 and table S2, Supplementary Material online) in these populations. First, we inferred admixture proportion in the admixed *groups* by averaging local ancestry inference (LAI) obtained with Loter (Dias-Alves et al. 2018). We divided derived mutations observed in N. American *groups* into three different categories according to SIFT4G (Kumar et al. 2009) predictions (see details in Materials and Methods) and calculated the frequency of these mutations in each *group* including the source *groups* (Hpg1 and non-Hpg1s). Derived alleles with frequency of 1 were considered as fixed, and based on the count we calculated fixed-to-total derived alleles ratios (ϕ) for each *group*. Considering admixture tracts of the diverged source lineages will result in slightly increased effective population sizes of the admixed groups, admixture is expected to reduce estimates of ϕ regardless of mutational categories. As genome-wide synonymous derived variation is expected to be effectively neutral with respect to fitness in small populations (Walsh and Lynch 2018), we considered ϕ_syn_ as a baseline for the reduction in ϕ due to admixture and divergence, and therefore scaled ϕ_non-syn-deleterious_ and ϕ_non-syn-tolerated_ with ϕ_syn_ (details in Materials and Methods). The scaled ϕ_non-syn-deleterious_ (*Φ*) is significantly lower (Welch’s *t-*test *P* value = 6.35e-12) than for nonsynonymous tolerated mutations (fig. 5 and supplementary fig. S18*C* and table S17, Supplementary Material online). Further, admixture had a strong effect on reducing *Φ*_non-syn-deleterious_, whereas *Φ*_non-synonymous-tolerated_ was not affected by it (supplementary fig. S18*B*, Supplementary Material online). The change from, *Φ*_non-syn-tolerated_ to *Φ*_non-syn-deleterious_ was significantly different between admixed and source *groups* (supplementary fig. S18*D*, Supplementary Material online). Similar patterns were observed when *Φ* was estimated using the total genome-wide ϕ for scaling across mutational categories (supplementary fig. S18*E*, Supplementary Material online). This strongly suggests that admixture helps to eliminate derived mutations with potential deleterious impacts and it efficiently reduces nonsynonymous mutational load.

### Ongoing Selection at Several Immunity Loci in N. America

Apart from admixture potentially reducing mutational load, it can also be a source of beneficial alleles. If such alleles are strongly selected, they will create signatures of a selective sweep (Smith and Haigh 1974; Stephan 2019; Moest et al. 2020). To look for such a scenario, we focused on large populations comprising several *groups* that apparently arose as a result of admixture between lineages that diverged before their introduction to N. America (supplementary fig. S3, Supplementary Material online). These populations were INRC (Indiana), NJSC (New Jersey), MISJ (Michigan), OHML, and OHPR (both Ohio).

**Fig. 6.**
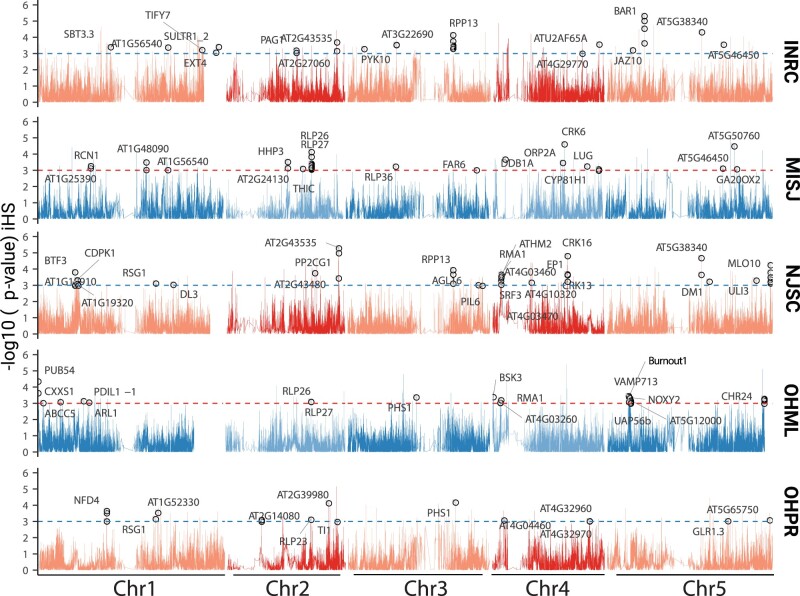
Genome-wide haplotype-based selection statistics in five N. American populations. Genome-wide *P* values of |*iHS*| scores (based on empirical distribution). Dashed horizontal lines correspond to a *P* value significance threshold of 0.001. Selection candidates, which also had |*n*S_L_| scores of >2, from the enriched GO categories of response to stress and response to stimulus (Fisher’s exact test with Bonferroni correction, *P* value <0.001 and FDR <0.05) are plotted with gene names or gene IDs.

Methods that track the decay of haplotype homozygosity in a population (Vatsiou et al. 2016) can be used to detect such sweeps. We scanned whole genomes for signals of natural selection using haplotype homozygosity-based tests *iHS* (integrated haplotype homozygosity score) (Voight et al. 2006) and *nS*_L_ (number of segregating sites-by-length) (Ferrer-Admetlla et al. 2014) for individual populations (supplementary tables S5–S9, Supplementary Material online) and *xp-EHH* (cross population extended haplotype homozygosity) (Sabeti et al. 2007) for comparisons between population pairs. For individual populations, we focused on variants with |*iHS*| *P* values for <0.001 and |*nS*_L_| values >2 (supplementary tables S5–S9, Supplementary Material online). GO-term analysis of the 82 genes tagged by these variants revealed an enrichment of genes in the categories “response to stress” and “response to stimulus” (*P* value after Bonferroni correction <0.001 and FDR <0.05) (fig. 6 and supplementary table S10, Supplementary Material online) We further confirmed this enrichment of GO-terms using a permutation method-based approach implemented in Gowinda (Kofler and Schlötterer 2012) (supplementary table S14, Supplementary Material online).

Consistent with the GO category enrichment, we noticed several NLR genes, a family that includes many known disease resistance genes (Van de Weyer et al. 2019). These included *RPP13 and BAR1*, which confer resistance to the oomycete *Hyaloperonospora arabidopsidis* (*Har*) (Bittner-Eddy et al. 2000) and bacteria of the genus *Pseudomonas* (Laflamme et al. 2020). We measured the frequency of alternative haplotypes around these loci to determine the nature of the selective sweep (Garud et al. 2015). The most frequent haplotype is designated as H_1_ and the second most as H_2_, from which a modified product of haplotype frequency (H_12_) and the H_2_/H_1_ ratio are calculated. At *RPP13 and BAR1*, we observed relatively low values for these two metrics and the presence of the selected alleles on multiple backgrounds, which together suggests soft sweeps at these loci (supplementary fig. S19*A* and *B*, Supplementary Material online). On the other hand, a pronounced hard sweep was observed in and around another putatively selected NLR, *BURNOUT1*, in the population OHML (supplementary fig. S19*A* and *B*, Supplementary Material online), with the selected allele found on a single haplotype. Similar to the |*iHS*| and |*nS*_L_| results, genes with high *xp-*EHH scores included several genes known to be involved in biotic and abiotic stress responses (supplementary table S11, Supplementary Material online).

In order to obtain direct evidence of whether the positively selected alleles are entering the population through admixture, we used data collected for presence of disease symptoms of downy mildew caused by *Har* (details in Supplementary Material online) on the samples. We chose the MISJ population for this analysis because the rate of infection in this population was comparatively higher than others with many RAD-seq genotyped individuals (36 diseased and 115 healthy individuals based on visual observation at the time of collection, supplementary table S15, Supplementary Material online). We observed that the genetic differentiation measured by *F*_ST_ was higher on chromosome 4 between groups of diseased and healthy individuals (supplementary fig. S20*A*, Supplementary Material online). MISJ has admixed individuals from Hpg1 and OhioMich1 source *groups* (fig. 2 and supplementary fig. S6 and table S2, Supplementary Material online), therefore we calculated *F*_ST_ of the two groups with Hpg1 separately. We found that the healthy individuals were significantly differentiated to Hpg1 in the genomic region of 9–12 Mb of chromosome 4 compared with the diseased individuals (supplementary fig. S20*B*, Supplementary Material online), whereas diseased individuals showed lower non-Hpg1 ancestry than the healthy individuals on chromosome 4 in general (supplementary fig. S20*C*, Supplementary Material online) and significantly lower non-Hpg1 local ancestry in the genomic region of 9–12 Mb (supplementary fig. S20*D* and *E*, Supplementary Material online). This genomic region is characterized by the presence of a well-known *Har* disease resistance gene cluster of *RPP4/5* (Noël et al. 1999; Holub 2001; van der Biezen et al. 2002). Thus, this result strongly suggests that *RPP4/5* genomic region from non-Hpg1 group OhioMich1 is preferentially kept in the MISJ population as it confers disease resistance.

## Discussion

How newly introduced, nonindigenous species adapt to new environments is a topic of long-standing interest in eco-evolutionary biology of invasive species (Baker and Stebbins 1965; Bock et al. 2015). There are two potential challenges facing invasive species: First, the niches in the new environment might be different from the ones in the native range and/or already filled by other species. Second, introductions typically begin with few individuals and therefore potentially a narrow genetic basis. The initial lack of genetic diversity can be overcome by new mutations or through the generation of new genetic combinations, either by crosses among the introduced population or with close relatives that are present in and already adapted to the new environment. We have used *A. thaliana* to address these questions.

*Arabidopsis thaliana* is native to Europe, Asia, and Africa, where it is found mostly as a human commensal (Hoffmann 2002; 1001 Genomes Consortium 2016; Durvasula et al. 2017; Lee et al. 2017; Zou et al. 2017). The human-assisted expansion of this species to N. America presents an excellent system to study processes associated with colonization of a new environment because it occurred recently and because the genetic diversity in the native range is so well documented for *A. thaliana*. Previous work has laid the groundwork for our study, but was limited by a paucity of genetic markers (Platt et al. 2010) or a focus on a single-dominant lineage (Exposito-Alonso, Becker, et al. 2018). We have investigated multiple individuals from several N. American populations at the whole-genome level, allowing us to describe fine-scale haplotype sharing within N. America and between N. America and individuals from the native range, either sequenced as a part of the 1001 Genomes project (1001 Genomes Consortium 2016) or subsequent efforts focused on Africa (Durvasula et al. 2017), China (Zou et al. 2017), and Ireland (this work).

### Multiple Independent Introductions

The extant diversity among *A. thaliana* individuals in N. America can be traced back to multiple, almost certainly independent introductions of lineages of diverged ancestries from three distinct geographic regions of Western Europe (British Isles/Ireland, Upper and Eastern France), central Europe (Germany, Czechia, and Austria), and Eastern Europe (the Baltic region and Russia). We detected these introductions using methods based on haplotype sharing (Lawson et al. 2012), allele frequencies (Patterson et al. 2012), and rare-allele sharing (Schiffels et al. 2016; Flegontov et al. 2019), lending considerable confidence to our findings and illuminating the extant diversity from several different angles. Significantly, even though we confirm that North-Western Europe and specifically the British isles are a major source of multiple introductions, the predominant lineage Hpg1, which has been estimated to have been introduced ∼400 years ago (Exposito-Alonso, Becker, et al. 2018), has more ancestry from Upper and Eastern France than from the British Isles. Its spread in N. America could be attributed to rapid expansion of French colonists from current Canada (then Acadia) along the Mississippi valley during the early period of post-Columbian colonization (Hamilton 1902). Our approach of haplotype-based clustering of individuals at different hierarchical levels using fineSTRUCTURE (Lawson et al. 2012) has allowed us to pinpoint several Western European sources of N. American *A. thaliana*. Although the sparse representation of individuals from Eastern Europe and Asia has limited our ability to more precisely identify the source of introductions from these regions, it is clear that Eastern Europe has contributed to extant N. American *A. thaliana* ancestry. Historical patterns of human migration indicate that northern and western Europeans arrived in significant numbers from the 1840s to 1880s, followed by waves of southern and eastern Europeans from the 1880s to 1910s (Passel and Fix 1994), which are reflected in the genetic make-up of present-day humans in N. America (Bryc et al. 2015; Dai et al. 2020). In the regions where we collected *A. thaliana* in N. America, humans have more British, Irish, central and eastern European ancestry than western, southern, and northern European ancestry (Bryc et al. 2015), consistent with the *A. thaliana* ancestry patterns. Thus, local anthropogenic introduction of *A. thaliana* can be proposed as a parsimonious explanation for the presence of diverged lineages in the regions of N. America that we sampled in our study.

### Wide-Spread Admixture

Perhaps our most significant finding is how multiple introductions have led to present-day N. American *A. thaliana* being surprisingly genetically diverse, different from many other colonizing or invading species (Dlugosch and Parker 2008). This highlights how between-population variation in the native range has translated into within-population variation in N. America (Rius and Darling 2014). In organisms with low outcrossing rates such as *A. thaliana*, benefits of local adaptation in the native range hinder admixture from other populations, even in the face of inbreeding depression. It has been argued that during invasion of new territory, there is a temporary loss of local adaptation that not only lifts the maladaptive burden of admixture but even favors admixture (Verhoeven et al. 2011; Rius and Darling 2014). We indirectly observe this in AEA *regions*’ geographically restricted haplotype-sharing patterns (supplementary fig. S24*C*, Supplementary Material online, quantified as Bray–Curtis distance in supplementary fig. S25, Supplementary Material online) whereas due to multiple introductions, N. American *A. thaliana* has a mixture of diverged ancestries (fig. 3*A*) and among some individuals levels of increased compositional dissimilarity (supplementary fig. S25, Supplementary Material online) similar to that seen in individuals from AEA *regions*. Further, compared with some AEA *regions*, nucleotide diversity (pi) and total derived allele count are elevated in a few N. American populations (supplementary fig. S26, Supplementary Material online). Patterns that are similar to the ones we have reported here for *A. thaliana* have been suggested for other systems, albeit mostly based on limited genetic information and without the benefit of being able to infer ancestry along each chromosome (Kolbe et al. 2004; Lavergne and Molofsky 2007; Facon et al. 2008; Smith et al. 2020).

Based on the observed lower selfing rates in N. America compared with Europe, it has been suggested that under slightly increased outcrossing, mixing of haplotypes should be expected (Platt et al. 2010). In line with this hypothesis, we observed that most N. American *A. thaliana* populations have individuals with admixture from the dominant Hpg1 *group*. Being apparently already well-adapted to the N. American ecological context upon its introduction, today Hpg1 is a wide-spread lineage in N. America (Platt et al. 2010; Exposito-Alonso, Becker, et al. 2018). Admixture with Hpg1, followed by selection, might have benefited and accelerated the spread of new incoming lineages. A case in support of this can be made for *group*s that are found in Indiana, where the human settlers in the mid-19th century came predominantly from North Carolina, Virginia, and Kentucky (Lynch 1915). Our demographic reconstructions using herbarium samples of SouthIndiana4 *group* estimated divergence times of 121 years-before present (126–119 HPD; 95%, supplementary fig. S21, Supplementary Material online) coincidental to the human migration and its admixture with Hpg1 has resulted in *groups* that are extant in North Carolina (our collection), Kentucky (1001 Genomes collection), and Georgia (herbarium collection). Alternative explanations such as short-term fitness benefits through heterosis (Facon et al. 2005; Keller and Taylor 2010) can currently not be ruled out, but could be tested with common garden experiments across N. American field sites. We also note that although some populations show richness in terms of haplotype diversity inferred with WGS (fig. 2) as a direct result of admixture events, our RAD-seq genotyping of over 2,000 individuals and coarse-scale haplotype ancestry and diversity estimates (supplementary figs. S22 and S23, Supplementary Material online) suggest that populations CTDE, CTPN, MAUR, and OHOS might have yet unexplored haplotype diversity.

### Purging of Deleterious Mutations

An important aspect of colonization is the severe genetic bottleneck due to founder effects and subsequent accumulation of deleterious mutations (Kirkpatrick and Jarne 2000; Verhoeven et al. 2011; Willi 2013; Schrieber and Lachmuth 2017), further exacerbated by predominant self-fertilization (Noël et al. 2017). One of the ways out of this invasion paradox (Estoup et al. 2016) might be admixture between colonizing lineages, which can both remove deleterious mutations (Heller and Maynard Smith 1978) and generate new genetic combinations that are only adaptive in the new environment (Dlugosch and Parker 2008; Rius and Darling 2014). Consistent with the expectation under admixture alone, we observed that the admixed N. American *A. thaliana* haplogroups have fewer fixed derived deleterious alleles. When background levels of reduction in fixed derived alleles using synonymous mutations were accounted for, we observed that compared with source *groups*, nonsynonymous tolerated mutations are removed at a lower rate than nonsynonymous deleterious mutations in the admixed *groups* (supplementary fig. S18*D*, Supplementary Material online). This demonstrates that admixture has been successful in removing some of the potential nonsynonymous mutational load carried by the founder lineages. A caveat is that the deleteriousness of variants is based on presumed reduction or loss of molecular function (Kono et al. 2018), even though gene inactivation can be adaptive as well (Olson 1999). A more direct approach to determining the extent of purging of mutational load in N. American colonizing lineages could come from direct estimates of local adaptation deficits and selection coefficients, by comparing the fitness of N. American individuals at their site of collection against a global sample of *A. thaliana* accessions (Exposito-Alonso et al. 2019) or by quantifying the amount of genetic rescue or F_1_ heterosis in crosses between populations (Koski et al. 2019).

### Resistance Genes as Loci under Selection

An indication of selection having potentially shaped the geographic distribution of genetic diversity in N. American *A. thaliana* is the observation of environmental dissimilarity between N. American haplogroups and their source lineages from the native range being negatively correlated with shared ancestry between them. Given that *A. thaliana* is a human commensal in its native range, it is not hard to envision that anthropogenically induced adaptation to invade (AIAI) (Hufbauer et al. 2012) might play a significant role in having accelerated *A. thaliana*’s adaptation to the N. American environment.

If a species is far from an adaptive peak, large-effect mutations are particularly likely to affect the speed of adaptation (Fisher 1930). Although the relative importance of abiotic and biotic factors for adaptation is still debated (Morris et al. 2020), some of the most drastic effects arise from disease resistance genes, where single genes have outsized effects on fitness and survival on plants in the presence of pathogens. In *Capsella*, it has been shown that dramatic losses of genetic diversity after extreme genetic bottlenecks can be tolerated at most genes in the genome, except for immunity loci (Koenig et al. 2019). Our selection scans with *A. thaliana* individuals from five different N. American populations have revealed that genes related to biotic stress are enriched among selection candidates. These include genes known to have alleles that confer resistance to two of the most prominent pathogens of *A. thaliana*, *H. arabidopsidis*, and *Pseudomonas* (Holub and Beynon 1997; Karasov et al. 2014, 2018). One of the loci we found to be under selection is *RPP13* (Rose et al. 2004), whose product recognizes the coevolved, highly polymorphic effector ATR13 from *H. arabidopsidis* (Allen et al. 2004). Another one is *BAR1*, whose product recognizes members from the conserved HopB effector family from *Pseudomonas* (Laflamme et al. 2020). Although *RPP13* is under balancing selection in at least part of the native range (Allen et al. 2004), we observe that a specific *RPP13* allele is found on different haplotypes (supplementary fig. S28, Supplementary Material online), has a comparable nucleotide diversity to AEA ancestral source *regions* (supplementary fig. S29, Supplementary Material online) and has undergone a selective sweep in N. American *A. thaliana* populations. Given that *H. arabidopsidis* appears to be an *A. thaliana* specialist (Slusarenko and Schlaich 2003), it must have been introduced with its *A. thaliana* host, and its genetic diversity in the introduced range might be as low or even lower than that of its host, potentially providing an explanation for the apparent selective sweep at *RPP13*. Apart from haplotype homozygosity-based scans, we observed high differentiation in the *RPP4/5* region of the genome between individuals in the MISJ population that were or were not visibly infected with *H. arabidopsidis* when we collected them. Local ancestry estimates confirm that this region of the genome has been entering the population through admixture and is of non-Hpg1 origin. As *RPP4/5* is known to harbor high levels of polymorphism and is known to be involved in frequency-dependent selection for resistance to *H. arabidopsidis* (Noël et al. 1999), this introgression event could be driven by positive selection against local strains of this pathogen.

## Conclusions

Altogether, our analysis using WGS from extant N. American *A. thaliana* has established a scenario of multiple introductions from sources of previously diverged Eurasian lineages. We provide evidence that new haplotype diversity has been generated through wide-spread admixture among introduced lineages, relieving mutational load and providing raw material for selection to act upon. Our findings are thus consistent with earlier proposals that hybridization can lead to the introduction of adaptive variation via introgression or admixture (Anderson 1948, 1949; Stebbins 1959; Grant 1981). The advent of molecular analyses has confirmed the relevance of hybridization for adaptation and speciation (Arnold 1996, 2004; Rieseberg 1997) and our observations are consistent with admixture being important for invasive success. Admixture can facilitate successful colonization when individuals from divergent populations have been recurrently introduced to a new range (Rius and Darling 2014; Dlugosch et al. 2015; Estoup et al. 2016). North American *A. thaliana* therefore may not have suffered from the genetic paradox of invasion (Allendorf and Lundquist 2003; Estoup et al. 2016). Finally, because *A. thaliana* has also colonized other continents, including S. America and Australia (Alonso-Blanco and Koornneef 2000; Kasulin et al. 2017), it will be interesting to determine both how genetic diversity of *A. thaliana* in these other places compares with N. America, and how genetic diversity of *A. thaliana* compares with that of other plants that have been inadvertently introduced to N. America by humans (Neuffer and Hurka 1999; Durka et al. 2005; La Sorte et al. 2007).

## Materials and Methods

### Sample Collection and Sequencing

Some samples were collected dried by pressing in acid-free paper with a wooden press for 8–12 weeks to produce herbarium samples. For other field samples, two to three well-expanded leaves were collected in a microcentrifuge tube and immediately placed on dry ice and kept at −80 °C until further processing. Seeds of Irish accessions were grown in the lab from seeds. Details of DNA extraction using different protocols and sequencing can be found in Supplementary Material online.

### Mapping and Variant Calling

Reads were mapped using bwa-mem (bwa-0.7.15) (Li and Durbin 2009) to the TAIR10 reference genome (https://www.arabidopsis.org/download_files/Genes/TAIR10_genome_release/TAIR10_chromosome_files/TAIR10_chr_all.fas, last accessed September 13, 2021) and sorted using samtools v1.3 (Li and Durbin 2009). Reads from herbarium samples were additionally trimmed with skewer (v. 0.1.127) (Jiang et al. 2014) using default parameters and merged with flash (v. 1.2.11) (Magoč and Salzberg 2011) with a maximum overlapping value of 150 bp, prior to mapping. SNP calling was performed with the Genome Analysis Tool Kit (GATK) best practices with modifications for single-end reads (DePristo et al. 2011; Van der Auwera et al. 2013). GATK tools used are described in supplementary methods, Supplementary Material online, and detailed parameters can be found in the script provided in the accompanying repository. Strategy and procedure to include SNPs from remaining *A. thaliana* global diversity data set can be found in the Supplementary Material online.

### Estimation of Recombination Rates

Haplotype phasing for estimation of recombination rate was performed with ShapeIt2 (v2.r837) (Delaneau et al. 2013) on samples from this project and a subset of the 1001 Genomes project (1001 Genomes Consortium 2016). After phasing, the recombination rate variation along the chromosomes was estimated using LDhelmet v1.7 (Chan et al. 2012). Detailed procedure is described in the supplementary methods, Supplementary Material online.

### Population Genetic Analysis

#### PCA, UMAP, and IBD

Principal component analysis was performed using SmartPCA of EIGENSOFT version 6.0.1 (Patterson et al. 2006) package. We used the first 50 PCs as input for generating two UMAP embeddings using Python package umap v0.4.6 (McInnes et al. 2018). Details of the analyses are in the supplementary methods, Supplementary Material online. Identity-by-descent and identity-by-state analyses were carried out with PLINK v1.90 (Chang et al. 2015).

#### Chromosome Painting and Clustering

Clustering of individuals based on shared ancestry from haplotype data was performed using fineSTRUCTURE on a coancestry matrix derived with the software CHROMOPAINTER v2 (Lawson et al. 2012), which treats all the individuals (except the individual whose ancestry is being reconstructed) as donor haplotypes and generates a mosaic of shared chunks copied from these donors in a given recipient individual. Similarity in the patterns of shared chunks (copying vectors) is indicative of shared ancestry and is the basis of the model-based clustering approach taken by the fineSTRUCTURE algorithm. Specifically, we performed this analysis in the following hierarchical way:

1. All N. American individual haplotypes were painted as a mosaic of all other N. American individuals’ haplotypes (self-excluding).
2. All non-N. American (Afro-Eur-Asian/AEA) haplotypes were formed as a mosaic of each other. Based on the haplotype sharing these individuals were then clustered and grouped into what we call *subclusters*, *clusters* (comprising *subclusters*), and *regions* (comprising clusters representing specific geographical regions).
3. All N. American haplotypes were then formed as a mosaic of AEA haplotype clusters. Detailed description of the analysis is in the supplementary methods, Supplementary Material online.

#### Treemix Analysis

We determined the phylogenetic relationship among the N. American *groups* and among these *groups* and AEA *subclusters* as inferred by fineSTRUCTURE using Treemix v1.13 (Pickrell and Pritchard 2012). Details are in the supplementary methods, Supplementary Material online.

#### f_3_-Outgroup Analysis

To determine the extent of shared drift between the AEA subclusters (smallest fineSTRUCTURE grouping) and N. American haplogroups, we used *f*_3_-outgroup tests as described (Patterson et al. 2012). *N. American_(i)_, AEA SubCluster_(j)_: Relicts (Fs12_3)* configuration was used and implementation of the test was carried out using R package “*admixr*” (Petr et al. 2019).

#### qpWave and D-Statistic Analyses

To determine the minimum number of ancestry waves from AEA regions (comprised different haplogroup *subclusters* defined by fineSTRUCTURE analyses), we used *D*-statistic and qpWave analyses from ADMIXTOOLS (Reich et al. 2012). Details of the tree configurations and outgroups can be found in the Supplementary Material online.

#### Rare Allele Sharing

About 1,039 AEA individuals that formed the fineSTRUCTURE *subclusters* were used as a reference panel to ascertain rare alleles and calculate RAS between AEA *subclusters* and N. American haplogroups. The input files were prepared with the tools from repository at (https://github.com/stschiff/rarecoal-tools, last accessed September 13, 2021) and the analysis was performed by the pipeline available at (https://github.com/TCLamnidis/RAStools, last accessed September 13, 2021). Minimum allele count of 2 and maximum allele count of 20 was used on the SNPs with less than 10% missing data. Alleles were polarized with the *A. lyrata* data.

#### Phylogenetic Methods

Bayesian phylogenetic analyses were carried out using BEAST v.2.4.8 (Bouckaert et al. 2014) for *groups* Hpg1 and SouthIndiana4. Details of the substitution model and prior used are in the Supplementary Material online.

#### Local Ancestry Inference

We performed LAI for RAD-seq genotyped samples from population MISJ and WGS samples from MISJ, NJSC, OHML, and OHPR with Loter model (Dias-Alves et al. 2018). Exact parameters and individuals used as reference source *groups* are described in the Supplementary Material online.

#### Genetic Differentiation in MISJ Population

We calculated genetic differentiation (*F*_ST_) between individuals that showed visible symptoms of infection by *H. arabidopsidis* (described in Koch and Slusarenko [1990]) at the time of collection and individuals were visibly healthy. Detailed strategy to determine the significance of the differentiation between the two groups is described in the Supplementary Material online.

### Environmental Factor Analysis

Historical climate data from 1970 to 2000 were downloaded from WorldClim2.0 (Fick and Hijmans 2017) at 2.5-min resolution using Python library latlon-utils 0.0.5 (https://github.com/Chilipp/latlon-utils, last accessed September 13, 2021). Environmental variables average temperature (°C), precipitation (mm), solar radiation (kJ m^−2 ^day^−1^), and water vapor pressure (kPa) were used for further analysis. Pairwise Euclidean distances of all the environmental variables were calculated for each N. American haplogroup to AEA *subclusters* (mean Latitude–Longitude of individuals in a given *subcluster* was used) and standardized values were used to model shared drift (measured by *f*_3_-outgroup statistics) among N. American haplogroups and AEA *subclusters* as a function of the environmental variables using Bayesian multilevel (hierarchical) linear regression. Description of the priors and hyper-priors is in the supplementary methods, Supplementary Material online.

Projection of the N. American haplogroups in reduced dimension formed by standardized average temperature, precipitation, and vapor pressure was performed using uniform manifold approximation and projection (UMAP) (McInnes et al. 2018). Two independent runs of UMAP were performed with different random numbers. In both the runs default “Euclidean” distance was used to compute distances in high dimensional space. We further used Ward’s linkage function on UMAP embeddings to determine hierarchical clustering patterns in the data set based on Euclidean distance. Details of the scripts and notebooks used for the analysis are in the accompanying repository.

### Estimation of Scaled Fixed-to-Total Derived Allele Ratio (*Φ*) for N. American *Groups* across Mutational Categories

Ancestral state of the positions was determined using pairwise alignments between *A. thaliana* and outgroups *A. halleri* and *A. lyrata* (ftp://ftp.ensemblgenomes.org/pub/plants/release-44/maf/ensembl-compara/pairwise_alignments/, last accessed September 13, 2021). The detailed strategy is described in the supplementary methods, Supplementary Material online. Precomputed SIFT 4G predictions for *A. thaliana* were obtained from (https://sift.bii.a-star.edu.sg/sift4g/public//Arabidopsis_thaliana/, last accessed September 13, 2021). Using these predictions, positions were divided into three different categories: 1) High-confidence deleterious mutations (score = 0–0.05), 2) Nonsynonymous tolerated mutations (score = 0.05–1) and, 3) synonymous mutations. We then calculated the ratio of fixed-to-total derived alleles ($\phi_{category}$) in every group separately for all categories (frequency of fixed alleles=1). We also calculated the genome-wide ratio of fixed-to-total derived alleles ($\phi_{\mathrm{genome}}$). ϕ is influenced by the increase in effective population size as a result of admixture between the source *groups* and divergence. To account for this in comparisons among *groups*, we scaled ϕ for high-confidence deleterious and for nonsynonymous tolerated mutations by dividing it with ϕ calculated for synonymous mutations for individual groups:

$$\Phi_{category}=\frac{\phi_{category}}{\phi_{syn-tol}}.$$

In addition to the scaling with synonymous variation we separately scaled the ratio with genome-wide fixed-to-total derived alleles ratio (using all the derived variation).

### Genome-Wide Selection Scans

We performed haplotype homozygosity-based selection scans to detect recent and ongoing selection. iHS (integrated haplotype score) (Voight et al. 2006) and XP-EHH (cross-population extended haplotype homozygosity) (Sabeti et al. 2007) were calculated using hapbin (Maclean et al. 2015), details are described in the supplementary methods, Supplementary Material online. Recombination map generated earlier was used in the estimation of both the statistics. $nS_{L}$ (number of segregating sites by length) (Ferrer-Admetlla et al. 2014), Garud’s H1, H12, and H2/H1 (Garud et al. 2015) (window size=500, step size=10), Tajima’s *D* (window size = 50,000 and step size = 5,000) were calculated with scikit-allel (Miles et al. 2020). Nucleotide diversity for the population was calculated using a pipeline described by (Martin et al. 2015).

$\left|\mathrm{iHS}\right|$ and $\left|nS_{L}\right|$ were used in a complementary manner. As iHS is known to be affected by recombination rate variation (O’Reilly et al. 2008), we used iHS first, and based on empirical distribution of the scores, *P* values were calculated per SNP. $nS_{L}$ was then calculated on the same data set. As $nS_{L}$ is robust to variation in mutation and recombination rates (Ferrer-Admetlla et al. 2014), overlap of the SNPs that showed $\left|\mathrm{iHS}\right|$*P* value less than 0.001 and$\left|nS_{L}\right|$ higher than 2 was taken as a signal of selection. GO-term analysis of the genes carrying the candidate selected SNPs was performed with AgriGOv2 (Tian et al. 2017) with PlantGo-Slim categories. For enrichment of GO terms, Fisher’s exact test with Bonferroni correction was used. We also performed GO-term enrichment analysis using a permutation-based method implemented in Gowinda v1.12 (Kofler and Schlötterer 2012) because it takes into account gene clustering and size.

## Supplementary Material

Supplementary data are available at *Molecular Biology and Evolution* online.

## Supplementary Material

msab268_Supplementary_Data
