# Supplementary material for: Multiple Sources of Introduction of North American Arabidopsis thaliana from across Eurasia
Source: Mol Biol Evol. 2021 Sep 9;38(12):5328–44. doi: 10.1093/molbev/msab268 (PMC8662644; doi:10.1093/molbev/msab268)
Supplement: msab268_Supplementary_Data [file msab268_supplementary_data.zip › Shirsekar2021_Supplementary_Material_R1.pdf]

Shirsekar et al. Multiple Sources of Introduction of North American *Arabidopsis thaliana* From Across Eurasia

Supplementary Material

Supplementary Methods

DNA extraction

Genomic DNA was extracted with the PTB method (Kistler 2012) for the dried leaf samples and with the method described (Clarke 2009) for 2-3 mg fresh tissue from well-expanded leaves from field or lab grown plants of Irish origin.

Sequencing

Libraries for reduced-representation (RAD-seq) with KpnI enzyme were prepared as described (Rowan et al. 2017) from 100 ng genomic DNA and sequenced to a coverage of 25-30x. Whole-genome sequencing (WGS) was performed with coverage of 12-15x (Karasov et al. 2018). Libraries were sequenced on Illumina HiSeq2000 (RAD-seq: 1 x 100 bp and 1 x 150bp single-end) and HiSeq3000 (WGS: 2 x 150bp paired-end) platforms. See Table S1 for sequencing metrics. Short reads (fastq) for selected *A. thaliana* from Africa (Durvasula et al. 2017), China (Zou et al. 2017), herbarium (Exposito-Alonso et al. 2018) and outgroup *A. lyrata* (Novikova et al. 2016) genomes were downloaded from NCBI-Sequence Read Archive using sratoolkit.2.8.2. Short reads of a subset of 145 accessions from the 1001 Genomes Project (1001 Genomes Consortium 2016) were downloaded from the servers at the MPI for Developmental Biology.

Tools used in GATKv3.5

GATKv3.5 pipeline was used on individual samples with the following suite of tools: RealignerTargetCreator, IndelRealigner, HaplotypeCaller, SelectVariants, VariantFiltration, BaseRecalibrator, PrintReads. Individual gvcf files were used for joint genotyping using GenotypeGVCFs tool of GATKv3.5 resulting in a merged variant call format (vcf) file.

Inclusion of SNPs from remaining A. thaliana global diversity dataset

Once the initial set of SNPs had been called from the samples sequenced in this project together with samples from the literature, additional SNP filtering was performed: 1. Bi-allelic; 2. missing less than 70% data; 3. Not from genomic regions annotated as transposable elements and 2,000 bp up and down-stream from these

annotations; 4. QD (Quality-by-depth) greater than 20. In total, 1,159,256 SNPs were retained. To include the remaining 990 individuals from 1,135 genomes, SNP calls from a publicly available SNP dataset (1001 Genomes Consortium 2016) were lifted from the 1,159,256 positions. This made some sites multi-allelic, and additional filtering was performed using vcftools v0.1.15 (Danecek et al. 2011) to retain only bi-allelic SNPs. SNPs with more than 10% missing data were discarded. The filtering strategy employed here resulted in a high-quality set of 862,934 SNPs with an average genotyping rate of 0.94 in 1,689 individuals.

Estimation of recombination rates

After haplotype phasing with ShapeIt2, a slightly different SNP and individual filtering strategy was used to avoid over-estimation of switch error rate (incorrectly phased heterozygous sites) because of the sensitivity of Shapeit2 to coverage and quality (Delaneau et al. 2013). Only individuals with average sequencing coverage (depth) >10x were kept. Then, the following filters were applied: 1. No singleton positions; 2. Not from genomic regions annotated as transposable elements and 2,000 bp up and down-stream from these annotations; 3. Not from NBS-LRR clusters with mean depth above 25. For LDhelmet v1.7, a mutation matrix was calculated using a parsimony-based method (Chan et al. 2012). In brief, if two samples of *A. lyrata* (outgroup) shared the same allele, it was assigned as ancestral and then these sites were used to calculate the mutation probability matrix. LDhelmet output is population scale recombination rate ($\rho = 4Ne \, . \, r$) in bp^{-1} units. For downstream analysis, we scaled this recombination rate in cM/Mb units by applying frequency-weighted means method (Booker et al. 2017) on an empirical recombination map of *A. thaliana* F_2 mapping populations (Salomé et al. 2012). The recombination map is available in the accompanying repository.

Principal Component Analysis (PCA) and uniform manifold approximation and projection (UMAP)

PCA: We used smartpca v.13050 for performing PCA on the merged RAD-seq and WGS datasets. Extremely diverged individuals of Relicts ancestry (Lee et al. 2017) affected the PCA strongly (PC1 and PC2 capturing differences between these individuals and the rest) and detailed population structure within the rest of the AEA individuals was not apparent in both the RAD-seq dataset (Fig. S2) and WGS. To overcome this, we used an outlier removal procedure (Option: numoutlieriter=2) with default parameters of outlier sigma threshold of 6.0 and number of PC components to perform outlier iterations equal to 10. For projection of N. American individuals into the PC space formed by the AEA individuals, we also used the above mentioned outlier removal procedure. We used individuals grouped into AEA *clusters* for the projection analysis and specified the list of these clusters in the 'poplistname' option in the smartpca parameter set-up file (this list is in the accompanying repository).

UMAP: For UMAP analysis we processed the genotype vcf file with the scikit-allel package (Miles et al. 2020) of Python. First, we performed LD-based pruning with 500 SNP sliding window size, 50 SNP as step size and r^2 threshold of 0.5, that resulted in retaining ~200,000 SNPs after 5 iterations . PCA was performed on this dataset using package scikit-learn v 0.23.2 (Pedregosa et al. 2011) . We used first 50 PC components for constructing two-dimensional embedding with number of neighbors =100 and minimum distance of 0.8 using Python package umap and umap-plot (McInnes et al. 2018) (details in the notebook umapWGS.ipynb in the repository). We also performed UMAP analysis on the co-ancestry matrix derived from the chromosome painting of AEA individuals that were subset using Identity-By-Descent (IBD) criteria with same number neighbors (100) and minimum distance parameter set to 0.8. UMAP embeddings on co-ancestry matrices derived by painting RAD-seq genotyped individuals of different N. American populations with AEA reference panel (described below in Chromosome painting and clustering section) were calculated by setting the following parameter values: 1. Number of neighbors = 50, min_dist = 0.5 and n_epochs = 100)

Chromosome painting and clustering

1. Generation of co-ancestry matrix with CHROMOPAINTER

Estimation of N_e and θ parameters*:* We ran CHROMOPAINTERv2 to estimate the nuisance parameters N_e and θ using the expectation-maximization option (100 steps). Runs for all 5 chromosomes were performed independently. Final N_e = 9838.359 and θ = 0.01126963 values were calculated by weight-averaging (across each chromosome by size in *cM*) as described in (Busby et al. 2015). Example command:

```
ChromoPainterv2 -g <chr_input _file> -t <individual_list> -a 0 0 -r <chr_recombination_file> -i 100 -in -iM -o <out_runA>
```

**Chromosome**	**N_SNPs**	N_e	θ
1	140484	11302.5	0.0110791
2	71243	6279.04	0.0111337
3	94479	14940.9	0.0116722
4	86040	6446.52	0.0120141
5	119015	8642.2	0.0107181

Table1: N_e and θ parameters for all the chromosomes

Building co-ancestry matrix: We generated two separate co-ancestry matrices using CHROMOPAINTER v2 runs on each chromosome separately for every Afro-EurAsian (AEA) individual (in AEA subset) and for every North American individual (in N. American set) using other individuals as donors in the respective

subsets for all the five chromosomes independently. Additionally, we derived two separate co-ancestry matrices after removing AEA individuals with IBD greater than 0.9 (PI_HAT statistic calculated with PLINK, which resulted in a set of 928 individuals). Two independent runs of CHROMOPAINTERv2 were conducted. Example command:

```
ChromoPainterv2 -g <chr_input _file> -t <individual_list> -a 0 0 -r <chr_recombination_file> -n 9838.359 -M 0.01126963 -o <chr_chunkcount>
```

Co-ancestry matrices of five chromosomes were combined into one matrix to be used for clustering, using following command:

```
fs chromocombine -o <all_chr_chunkcount>  <.chr*.RunX.chunkcounts.out>
```

2. Clustering with fineSTRUCTURE

Identification of clusters from the individuals: We used output of co-ancestry matrix for further model-based Bayesian clustering of individuals in the groups using fineSTRUCTURE (Lawson et al. 2012). One million iterations were run with ten thousand iterations as burn-in and sampling was performed every ten thousand iterations. We performed 3 such independent runs by setting different seed (-s option) generated by random numbers. Example command:

```
fs fs -X -Y -s $RANDOM -x 10000 -y 1000000 -z 10000 <all_chr_chunkcount> <mcmc_out>
```

Following the clustering we produced a tree that summarized the relationships between the individuals classified in different clusters. We considered upto ten million trees for comparisons for splitting or merging individuals from the clusters. Again, three separate runs on previously generated outputs from three independent mcmc runs for clustering were performed. Example command:

```
fs fs -X -Y -s $RANDOM -x 10000 -m T -t 10000000 <all_chr_chunkcount> <mcmc_out>  <mcmc_tree_out>
```

After each run final MAP states and mean coincidence for the tree file was generated using following example commands:

```
fs fs -X -Y -e X2 <all_chr_chunkcount> <mcmc_tree_out> <mcmc_mapstate.csv>
fs fs -X -Y -e meancoincidence <all_chr_chunkcount> <mcmc_out>  <mcmc_meancoincidence.csv>
```

Hierarchical ordering of clusters: We grouped individuals from AEA subset into 158 clusters that we call *sub-clusters* by visually inspecting the trees and merged these *sub-clusters* into 21 major clades that we call *clusters*. We then compared trees constructed with all the AEA individuals and trees constructed with co-ancestry matrices derived from AEA individuals with IBD not greater than 0.9. After visual inspection of overall consistency between these trees we further split these 21 major clades into 25 *regions* based on the geographical origin of the individuals in the *clusters*. The membership of individuals in these different *regions* is described in Table S3. Similar strategy of visually inspecting tree was used to group N. American

individuals into 58 clusters that we call *groups* (described in the Table S3). The *groups* were not further merged into clades.

3. Chromosome painting of individuals of N. American *groups* and subset of AEA individuals with an equal number of representative individuals of AEA *regions.*

We randomly chose 15 individuals from each AEA *region* as representatives (Table S12). Using this palette, we applied CHROMOPAINTERv2 's ability to infer haplotype sharing among individuals to estimate N. American and rest of the AEA individuals' copying profiles from the representative individuals of various AEA regions, independently. We set each N. American *group* (composed of member individuals) as a recipient and specified every individual from representative AEA-set as a donor according to its membership to a *region*. Thus , we specifically looked at haplotype chunks donated by AEA *regions* to N. American *groups* as a summary of haplotype segments donated by AEA individuals to N. American individuals. Similarly, every *sub-cluster* of remaining AEA individuals was set as a recipient and a haplotype copying profile of these individuals from the representative AEA individuals was built. Following example command was used to accomplish this:

```
ChromoPainterv2 -g <chr_input_phasefile> -t <group_membership> -f <donor_recipient_list> -r <chr.recombfile>
-n 9838.359 -M 0.01126963 -o <out_painting>
```

4. Chromosome painting of N. American individuals from different populations using RAD-seq genotypes with a subset of AEA individuals with an equal number of representative individuals of AEA *regions.*

Using the same set of representative individuals of different AEA *regions* we performed CHROMOPAINTERv2 analysis to determine the copying profiles of individuals that were sequenced with RAD-seq (total 20,233 markers) to understand the general patterns of ancestry in different N. American populations. The example command is similar to the one above in section 3.

5. Compositional dissimilarity

With the painting profiles of N. American and AEA individuals derived using representative subset of AEA individuals from different *regions* we calculated compositional dissimilarity using Bray-Curtis distance metric. We also calculated pairwise geographic distance (Haversine distance) between AEA *regions* and N. American *groups* for which the pairwise Bray-Curtis distances were calculated. The script can be found in the accompanying repository.

Treemix analysis

Treemix infers the relationship among populations as a graph structure derived from genome-wide allele frequency and genetic drift modeled as Gaussian distribution (Pickrell and Pritchard 2012). After processing the dataset with helper scripts available in the repository https://bitbucket.org/nygcresearch/treemix/wiki/Home , we used *A. lyrata* individuals as outgroup and set blocks of 100 SNPs to calculate the maximum likelihood tree using 6 bootstraps using Treemix v1.13.

qpWave and D-statistic analysis

qpWave and D-statistic analysis was carried out using ADMIXTOOLS (Reich et al. 2012). The analyses were performed in a hierarchical way. First, a set of AEA regions was chosen as outgroups (outgroup_layer1) that were donating the haplotype chunks at equal proportions to the analyzed N. American haplogroups. All the N. American haplogroup pairs were analyzed with this set of outgroups (*N. American1, N. American2:Outgroup1, Outgroup2*). Composition of this outgroup set is described in Table S4. The population pairs for which a Rank 0 matrix was accepted were considered to be from a single stream of ancestry compared to the outgroups (*p*-value >0.05). These pairs were then further analyzed one-by-one in another round of qpWave analysis with the same outgroup set plus one more *region* with higher contribution of haplotype chunks across the North American haplogroups (Fig. 3). These additional regions were: 1. BritishIsles2; 2. Italy/Balkan Peninsula; 3. NorthGermany; 4. RussiaAsia; 5. Upper/EastFrance BritishIsles.

Phylogenetic analyses with BEAST v.2.4.8

Bayesian phylogenetic analyses were carried out for the *groups* Hpg1 (N = 16) and SouthIndiana4 (N = 9). We restricted the analyses to biallelic homozygous SNPs, allowing a 10% of missing data, for a total number of 851,817 and 853,532 SNPs for Hpg1 and SouthIndiana4. In order to translate convergence relative times into calendar years, we used the accession collection dates to calibrate the tips (Table S13). We selected a general time-reversible substitution model (Abadi et al. 2019). Further, in order to reduce the effect based on demographic history assumptions, we used the Coalescent Extended Bayesian Skyline as prior (Drummond et al. 2005). For each population, we combined the output of four independent Monte Carlo Markov Chains, each of which had a length of 10 million iterations after which a maximum clade credibility (MCC) tree per *group* was computed. MCC trees were illustrated using FigTree v1.4.4 (http://tree.bio.ed.ac.uk/software/figtree/).

Local ancestry inference (LAI)

We estimated LAI-guided global average ancestry estimates in populations INRC, MISJ, NJSC and OHPR using a phased WGS SNP dataset (described in SNP calling section). We used reference individuals belonging to *group* Hpg1 and non-Hpg1 *groups* (SouthIndiana4 for INRC , OhioMich1 for MISJ and Ohio7,8 for OHPR). Details of the membership of individuals is in Table S3 . While running Loter on these samples a regularization parameter (λ) range of 1.5 to 5.5 with increments of 0.5 was used. Bagging with 40 different solutions (vote parameter = 0.75 and smoothing parameter = 0.9) was used to improve the stability of the solution for λ.

For MISJ individuals sequenced using RAD-seq approach, we prepared a subset of samples with metadata on visible *Hyaloperonospora arabidopsidis* (*Har*) infection with following additional filtering: 1.mean depth of sequencing 10x or above 2. SNP positions with less than 20% missing data, and 3. Individuals with less than 10% missing data. Using this strategy, we retained 36 individuals with visible *Har* infection and 115 without visible *Har* infection with a total of 93,687 SNPs on 5 chromosomes (marker density of 7.9 per 10 kb). We used individuals of source *groups* (Hpg1 and OhioMich1) identified using WGS and fineSTRUCTURE approach as a reference panel (Table S16).

Genetic differentiation in MISJ population

We used 36 individuals with visible *Har* infection and 115 with no visible *Har* infection with the same set of SNPs as used for LAI in these individuals for testing genetic differentiation along the genome. We first calculated F_{ST} in windows of 500kbp with 50kbp step size using vcftools v0.1.15 (Danecek et al. 2011). To observe the consistency of the estimates of F_{ST} we performed 1000 bootstraps with 2/3rd individuals from each group. To estimate the overall differentiation in the MISJ population we randomly assigned these individuals to two groups of the same size (36 and 115 individuals) and performed 1000 permutations. Further, we calculated genetic differentiation between Hpg1 individuals of the MISJ population and the two groups with the same window and step size.

Ancestral allele determination

Pairwise alignments between *A. thaliana* (TAIR10) and *A. halleri* and *A. lyrata* were converted to BAM format using maf-convert tool(Kiełbasa et al. 2011) and samtools(Li et al. 2009) was used to convert the alignment from BAM format to a VCF file which carried variant information. If both the *A. halleri* and *A. lyrata* had the same homozygous reference allele (with respect to TAIR10) at a given position, then that allele was considered ancestral. In case of alternate alleles, either the homozygous alternate alleles in both *A. halleri* and *A. lyrata* were considered as ancestral, or ; if there were homozygous alternate alleles in one and

heterozygous ones in the other, the consensus was considered to be ancestral (scripts and ancestral states are in the accompanying repository).

Environmental factor analysis

Bayesian Multi-level linear regression model (outgroup f_3) statistics as a function of environmental variables)

We modeled outgroup f_3 as a Student's T-distributed variable for *i*-th N. American *group* with normality parameter ν_i, location parameter μ_i and a global scale parameter σ

$$f_{3_i} \sim T(\nu, \mu_i, \sigma)$$

μ_i is expressed as a linear combination of standardized environmental factor dissimilarity of the *i*-th N. American *group* with all the 158 AEA *sub-clusters* with corresponding β coefficients.

$$\mu_i = \alpha_i + \beta_{t_i} tavg_i + \beta_{p_i} prec_i + \beta_{s_i} srad_i + \beta_{v_i} vapr_i$$

Hyper-priors: We set the following population wide hyper-priors.

$\bar{\alpha} \sim N(0,1) \rightarrow$ intercept
$\bar{\beta_t} \sim N(0,1) \rightarrow$ tavg coefficient
$\bar{\beta_p} \sim N(0,1) \rightarrow$ prec coefficient
$\bar{\beta_s} \sim N(0,1) \rightarrow$ srad coefficient
$\bar{\beta_v} \sim N(0,1) \rightarrow$ vapr coefficient
$\sigma \sim Exponential(1) \rightarrow$ scale parameter
$\nu \sim \gamma(\alpha = 2, \beta = 0.1) \rightarrow$ normality parameter

Priors: We specified all the group-specific priors as normally distributed

$$\alpha_i \sim N(\bar{a}, \sigma)$$
$$\beta_{t_i} \sim N(\bar{\beta_t}, \sigma)$$
$$\beta_{p_i} \sim N(\bar{\beta_p}, \sigma)$$
$$\beta_{s_i} \sim N(\bar{\beta_s}, \sigma)$$
$$\beta_{v_i} \sim N(\bar{\beta_v}, \sigma)$$

Implementation: Implementation of the model was carried out in python using the probabilistic programming package PyMC3 (v 3.9.3) (Salvatier et al. 2016). For the analysis we sampled 4 chains with 1000 iterations for tuning and 4000 iterations as draws. Jupyter notebook (outGroupF3_hierarchicalRegression.ipynb) and related python scripts used to import and format the data are available in the accompanying repository.

Projection of groups in reduced environmental space using UMAP: Bioclim data on the variables tavg, prec and vapr for each accession was downloaded according to its geographic location. We calculated the mean of each variable for every individual and then according to the individuals' membership to either AEA *sub-cluster* or N. American *group*, we grouped the individuals and calculated the average for that *sub-cluster* or *group*. The projection of the *sub-clusters* and *groups* in reduced environmental space was performed with

the python package umap-learn v0.4.6 (McInnes et al. 2018). umap_fs_newGroup_envVariableSpace.ipynb notebook in the repository describes the implementation.

Genome-wide selection scans

We applied haplotype homozygosity based statistics to scan the genomes of individuals from select N. American populations (INRC, MISJ, NJSC, OHML and OHPR). iHS (integrated haplotype score) (Voight et al. 2006) calculates extended haplotype homozygosity (EHH) among given genomes while taking into account local recombination rates. Standardized iHS scores can be calculated by considering SNPs with similar allele frequencies (Walsh and Lynch 2018). We calculated iHS for every population with hapbin (Maclean et al. 2015) where we set minimum allele frequency of 0.01 and EHH cutoff of 0.1 with other default parameters. Example command is:

```
ihsbin --hap <pop.chr_no.impute.hap --map pop.chr_no.impute.map --minmaf 0.01 --cutoff 0.1 --out pop.chr_no.ihs2
```

To complement the results from iHS we used standardized nS_L (number of segregating sites) (Ferrer-Admetlla et al. 2014) as implemented in Python package scikit-allel (Miles et al. 2020). nS_L is conceptually similar to iHS but instead of relying on local recombination rates, it relies on the number of adjacent polymorphic sites shared by a pair of haplotypes around focal SNP. Garud's H1,H12 and H2/H1 (in 500 SNPs window with step size of 10 SNPs) were calculated with the same package (implementation is described in the notebook nsl_GarudH_HaplotypeDiv_final.ipynb in the accompanying repository). As selected allele approaches fixation, it becomes harder for iHS and nS_L to detect the signal of selective sweep. If the selected allele is fixed in one population and not in the other,then between-population comparison still can identify the signature of selective sweeps (Walsh and Lynch 2018). Therefore, we applied the cross-population extended haplotype homozygosity (XP-EHH) test implemented in hapbin with default parameters. Example command:

```
xpehhbin --hapA <pop1.chr.impute.hap --hapB <pop2.chr.impute.hap> --map <chr.impute.map> --out <pop1vpop2.chr.xpehh
```

References

1001 Genomes Consortium. 2016. 1,135 Genomes Reveal the Global Pattern of Polymorphism in Arabidopsis thaliana. *Cell* 166:481–491.

Abadi S, Azouri D, Pupko T, Mayrose I. 2019. Model selection may not be a mandatory step for phylogeny reconstruction. *Nat. Commun.* 10:934.

Booker TR, Ness RW, Keightley PD. 2017. The Recombination Landscape in Wild House Mice Inferred Using Population Genomic Data. *Genetics* 207:297–309.

Busby GBJ, Hellenthal G, Montinaro F, Tofanelli S, Bulayeva K, Rudan I, Zemunik T, Hayward C, Toncheva D, Karachanak-Yankova S, et al. 2015. The Role of Recent Admixture in Forming the Contemporary

West Eurasian Genomic Landscape. *Curr. Biol.* 25:2518–2526.
Chan AH, Jenkins PA, Song YS. 2012. Genome-wide fine-scale recombination rate variation in Drosophila melanogaster. *PLoS Genet.* 8:e1003090.
Clarke JD. 2009. Cetyltrimethyl ammonium bromide (CTAB) DNA miniprep for plant DNA isolation. *Cold Spring Harb. Protoc.* 2009:db.prot5177.
Danecek P, Auton A, Abecasis G, Albers CA, Banks E, DePristo MA, Handsaker RE, Lunter G, Marth GT, Sherry ST, et al. 2011. The variant call format and VCFtools. *Bioinformatics* 27:2156.
Delaneau O, Howie B, Cox AJ, Zagury J-F, Marchini J. 2013. Haplotype estimation using sequencing reads. *Am. J. Hum. Genet.* 93:687–696.
Drummond AJ, Rambaut A, Shapiro B, Pybus OG. 2005. Bayesian Coalescent Inference of Past Population Dynamics from Molecular Sequences. *Mol. Biol. Evol.* 22:1185–1192.
Durvasula A, Fulgione A, Gutaker RM, Alacakaptan SI, Flood PJ, Neto C, Tsuchimatsu T, Burbano HA, Xavier Picó F, Alonso-Blanco C, et al. 2017. African genomes illuminate the early history and transition to selfing in Arabidopsis thaliana. *Proc. Natl. Acad. Sci. U. S. A.* 114:5213–5218.
Exposito-Alonso M, Becker C, Schuenemann VJ, Reiter E, Setzer C, Slovak R, Brachi B, Hagmann J, Grimm DG, Chen J, et al. 2018. The rate and potential relevance of new mutations in a colonizing plant lineage. *PLoS Genet.* 14:e1007155.
Ferrer-Admetlla A, Liang M, Korneliussen T, Nielsen R. 2014. On detecting incomplete soft or hard selective sweeps using haplotype structure. *Mol. Biol. Evol.* 31:1275–1291.
Karasov TL, Almario J, Friedemann C, Ding W, Giolai M, Heavens D, Kersten S, Lundberg DS, Neumann M, Regalado J, et al. 2018. Arabidopsis thaliana and Pseudomonas Pathogens Exhibit Stable Associations over Evolutionary Timescales. *Cell Host Microbe* 24:168–179.e4.
Kiełbasa SM, Wan R, Sato K, Horton P, Frith MC. 2011. Adaptive seeds tame genomic sequence comparison. *Genome Res.* 21:487–493.
Kistler L. 2012. Ancient DNA Extraction from Plants. In: Shapiro B, Hofreiter M, editors. Ancient DNA: Methods and Protocols. Totowa, NJ: Humana Press. p. 71–79.
Lawson DJ, Hellenthal G, Myers S, Falush D. 2012. Inference of population structure using dense haplotype data. *PLoS Genet.* 8:e1002453.
Lee C-R, Svardal H, Farlow A, Exposito-Alonso M, Ding W, Novikova P, Alonso-Blanco C, Weigel D, Nordborg M. 2017. On the post-glacial spread of human commensal Arabidopsis thaliana. *Nat. Commun.* 8:14458.
Li H, Handsaker B, Wysoker A, Fennell T, Ruan J, Homer N, Marth G, Abecasis G, Durbin R, 1000 Genome Project Data Processing Subgroup. 2009. The Sequence Alignment/Map format and SAMtools. *Bioinformatics* 25:2078.
Maclean CA, Chue Hong NP, Prendergast JGD. 2015. hapbin: An Efficient Program for Performing Haplotype-Based Scans for Positive Selection in Large Genomic Datasets. *Mol. Biol. Evol.* 32:3027–3029.
McInnes L, Healy J, Melville J. 2018. UMAP: Uniform Manifold Approximation and Projection for Dimension Reduction. *arXiv [stat.ML]* [Internet]. Available from: http://arxiv.org/abs/1802.03426
Miles A, Bot P io, Rodrigues MF, Ralph P, Harding N, Pisupati R, Rae S. 2020. cggh/scikit-allel: v1.3.1. Available from: https://zenodo.org/record/3935797
Novikova PY, Hohmann N, Nizhynska V, Tsuchimatsu T, Ali J, Muir G, Guggisberg A, Paape T, Schmid K, Fedorenko OM, et al. 2016. Sequencing of the genus Arabidopsis identifies a complex history of nonbifurcating speciation and abundant trans-specific polymorphism. *Nat. Genet.* 48:1077.
Pedregosa F, Varoquaux G, Gramfort A, Michel V, Thirion B, Grisel O, Blondel M, Prettenhofer P, Weiss R, Dubourg V, et al. 2011. Scikit-learn: Machine Learning in Python. *J. Mach. Learn. Res.* 12:2825–2830.
Pickrell JK, Pritchard JK. 2012. Inference of population splits and mixtures from genome-wide allele frequency data. *PLoS Genet.* 8:e1002967.
Reich D, Patterson N, Campbell D, Tandon A, Mazieres S, Ray N, Parra MV, Rojas W, Duque C, Mesa N, et al. 2012. Reconstructing Native American population history. *Nature* 488:370.
Rowan BA, Seymour DK, Chae E, Lundberg DS, Weigel D. 2017. Methods for Genotyping-by-Sequencing. In: White SJ, Cantsilieris S, editors. Genotyping: Methods and Protocols. New York, NY: Springer New

York. p. 221–242.
Salomé PA, Bomblies K, Fitz J, Laitinen RAE, Warthmann N, Yant L, Weigel D. 2012. The recombination landscape in Arabidopsis thaliana F2 populations. *Heredity* 108:447–455.
Salvatier J, Wiecki TV, Fonnesbeck C. 2016. Probabilistic programming in Python using PyMC3. *PeerJ Comput. Sci.* 2:e55.
Voight BF, Kudaravalli S, Wen X, Pritchard JK. 2006. A map of recent positive selection in the human genome. *PLoS Biol.* 4:e72.
Walsh B, Lynch M. 2018. Evolution and Selection of Quantitative Traits. Oxford University Press
Zou Y-P, Hou X-H, Wu Q, Chen J-F, Li Z-W, Han T-S, Niu X-M, Yang L, Xu Y-C, Zhang J, et al. 2017. Adaptation of Arabidopsis thaliana to the Yangtze River basin. *Genome Biol.* 18: 239.

List of Supplementary Tables

Table S1. Provenance and sequencing depth of individuals

TableS2. Scores of *f3*-statistic to test admixture among N. American *groups* in the form (*groupA*,*groupB*:*testGroup*) where z-scores are less than -3.0

Table S3. List of AEA sub-clusters and N. American *groups* and their member individuals

Table S4. Results of *qp-Wave* analysis

Table S5. |*iHS*| and |*nSL*| scores for SNPs showing significant |*iHS*| values in the population **NJSC** and gene IS of the SNPs

Table S6. |*iHS*| and |*nSL*| scores for SNPs showing significant |*iHS*| values in the population **MISJ** and gene IS of the SNPs

Table S7. |*iHS*| and |*nSL*| scores for SNPs showing significant |*iHS*| values in the population **OHPR** and gene ID of the SNPs

Table S8. |*iHS*| and |*nSL*| scores for SNPs showing significant |*iHS*| values in the population **OHML** and gene ID of the SNPs

Table S9. |*iHS*| and |*nSL*| scores scores for SNPs showing significant |*iHS*| values in the population **INRC** and gene ID of the SNPs

Table S10. GO term enrichment analysis for |iHS| significant SNPs (*p*-value less than 0.001)

Table S11. *xpEHH* values of SNPs and their *p*-values in cross-population comparisons along with the IDs of genes carrying these SNPs and their GO-description

Table S12. Reference panel of AEA individuals used for chromosome painting

Table S13. Collection years of the accessions used in the Bayesian phylogenetic analysis with BEAST

Table S14A. GO term enrichment analysis for significant SNPs in the population INRC based on |iHS| and |nSL| statistics with Gowinda (FDR < 0.1)

Table S14B. GO term enrichment analysis for significant SNPs in the population MISJ based on |iHS| and |nSL| statistics with Gowinda (FDR < 0.1)

Table S14C. GO term enrichment analysis for significant SNPs in the population NJSC based on |iHS| and |nSL| statistics with Gowinda (FDR < 0.1)

Table S14D. GO term enrichment analysis for significant SNPs in the population OHML based on |iHS| and |nSL| statistics with Gowinda (FDR < 0.1)

Table S14E. GO term enrichment analysis for significant SNPs in the population OHPR based on |iHS| and |nSL| statistics with Gowinda (FDR < 0.1)

Table S15. Metadata on MISJ individuals and reference panel used for LAI using Loter

Table S16. Individuals in different populations used for LAI using Loter

Table S17. Derived allele counts in different mutational categories and scaled fixed-to-total derived allele ratio for N. American *groups*

Download of supplementary tables:

https://nextcloud.tuebingen.mpg.de/index.php/s/kamQHKagwZjX7y9

List of Supplementary Figures

Figure **Page**

S1. Population structure and genetic similarity of N. American populations in the context of global populations s16

S2. PCA using RAD-seq SNP markers with no outlier iterations implemented in SmartPCA s17

S3. Identification of different haplogroups in N. American individuals s18

S4. Maximum likelihood tree of N. American groups using Treemix s19

S5. Shared drift among N. American haplogroups using f_3 outgroup analysis s20

S6. Admixed groups and shared drift of Haplogroup1 with other haplogroups s21

S7. fineSTRUCTURE clustering of non-American *A. thaliana* individuals from the native range and chromosome painting of N. American groups with the *regions* as donors s22

S8. Haplotype copying profiles of different N. American haplogroups with AEA reference panel s23

S9. Shared drift of N. American haplogroups with AEA sub-clusters with f_3 outgroup analysis s24

S10. Maximum likelihood tree of North American groups and AEA *sub-clusters* s25

S11. N. American and AEA individuals in reduced dimensions s27

S12. Rare allele sharing of N. American haplogroups with AEA sub-clusters s28

S13. Posterior means and 89% compatibility interval for individual group's β coefficients for tavg (bT), precipitation (bP), solar radiation (bS) and water vapor pressure (bV) s29

S14. Sampled posterior regression lines for the model describing relationship between f_3-outgroup statistics and environmental variables s30

S15. Trace plots for Bayesian Multi-level Model (bMLM) s31

S16. Projection of populations consisting of N. American *groups* and AEA *sub-clusters* in reduced environmental dimensions s32

S17. Hierarchical clustering on reduced environmental space of N. American *groups* and AEA *regions* s33

S18. Fixed derived alleles to the total derived alleles ratio for the N. American *groups* s34

S19. Haplotype homozygosity based statistics on three NBS-LRR domain containing genes s36

S20. Differentiation between infected and non-infected individuals of the population MISJ s37

S21. Maximum Clade Credibility (MCC) trees of *group* Hpg1 and SouthIndiana4 s39

S22. Population wide haplotype sharing of RAD-seq genotyped North American individuals s40

S23. Genetic variation in the RAD-seq genotyped individuals s41

S24. Ancestry profiles of AEA *regions* subset individuals s42

S25. Compositional dissimilarity in AEA and N. American individuals s43

S26. Measure of diversity in AEA *regions* and N.American populations s44

S27. xpEHH (cross-population extended haplotype homozygosity) scores' *p*-values among all the population comparisons (*y*-axis is -log10(*p*-value)) on Chromosome 3 s45

S28. Comparison of selected SNP's haplotype homozygosity block with a nearby SNP with similar allele frequency s46

S29. Nucleotide diversity (π) on chromosome 3 in AEA *clusters* and N. American populations s47

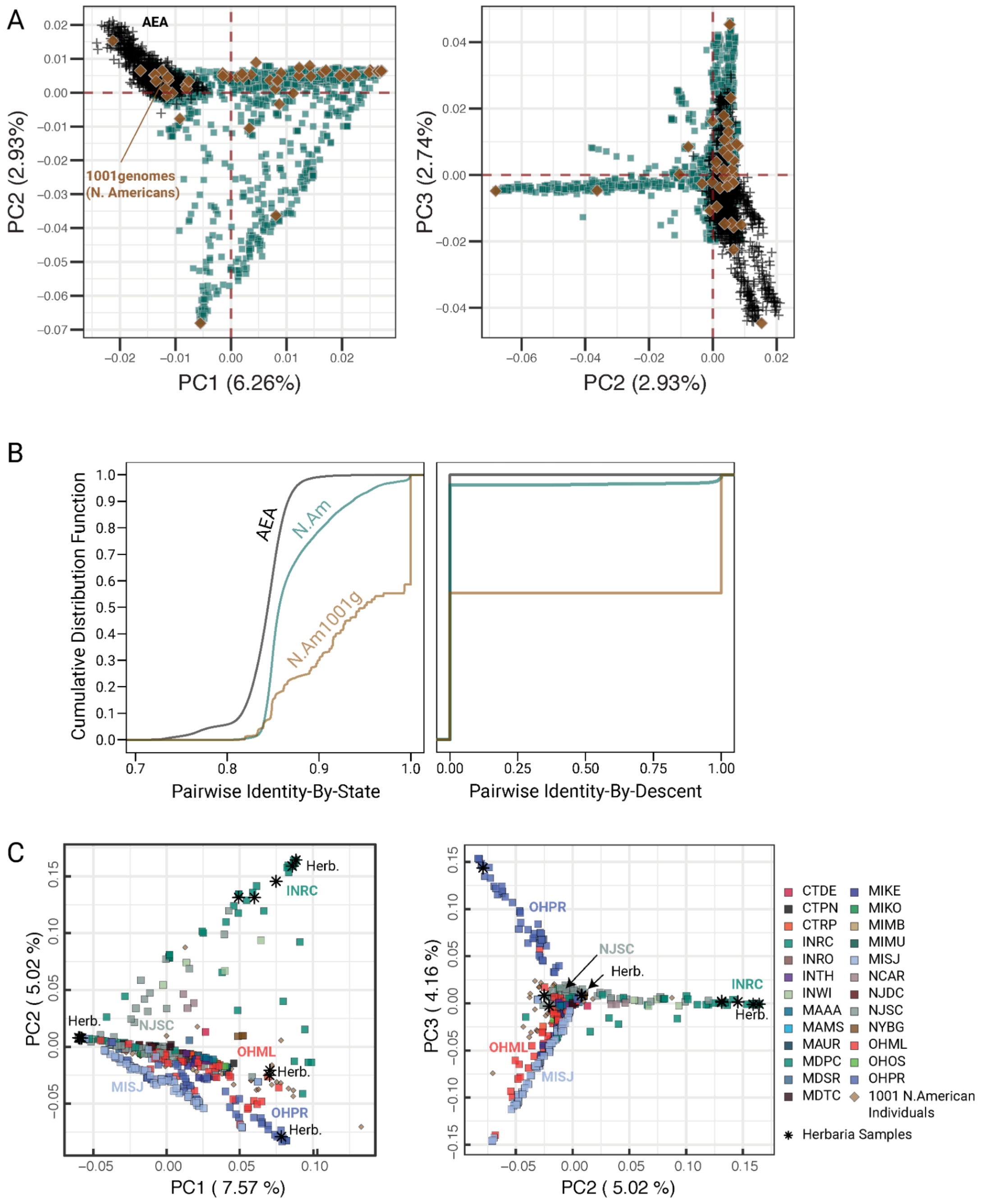

Figure S1. Population structure and genetic similarity of N. American populations in the context of global populations

A. Principal component analysis (PCA) with ~5,000 RAD-seq SNP markers with five outlier iterations in Eigenstrat SmartPCA. N. American individuals from this study in blue (n = 3,232). Brown: N. American individuals from 1001 Genomes (1001G) collection (n =135). Black: Individuals from Afro-Eurasia (AEA, n=1,194). **B.** Empirical cumulative density function of genetic similarity estimated using pairwise Identity-by-State (DST) and Identity-by-descent among N. American (blue) individuals, N. American (1001G individuals) and AEA individuals (black). **C.** PCA using whole genome sequences of 500 individuals (sampled in this work plus 1001G N. American individuals plus herbaria individuals) with ~900,000 SNPs.

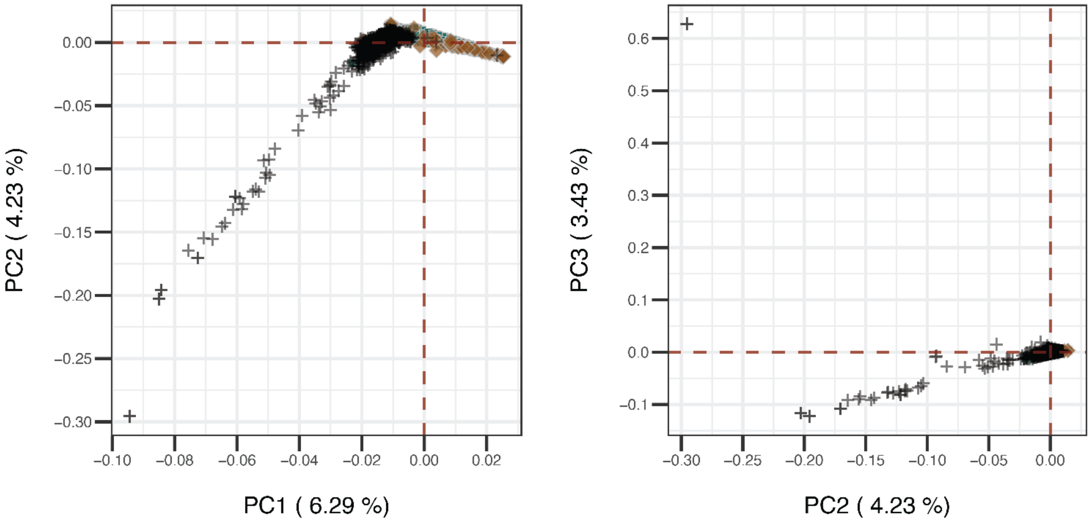

Figure S2. PCA using RAD-seq SNP markers with no outlier iterations implemented in SmartPCA
Blue, individuals collected in this work; brown: N. American individuals from 1001 Genomes collection (n=135); black: Afro-Eur-Asian individuals (n=1,194). Outliers detected here are individuals from relict populations from Iberian Peninsula, Sicily and sub-Saharan Africa.

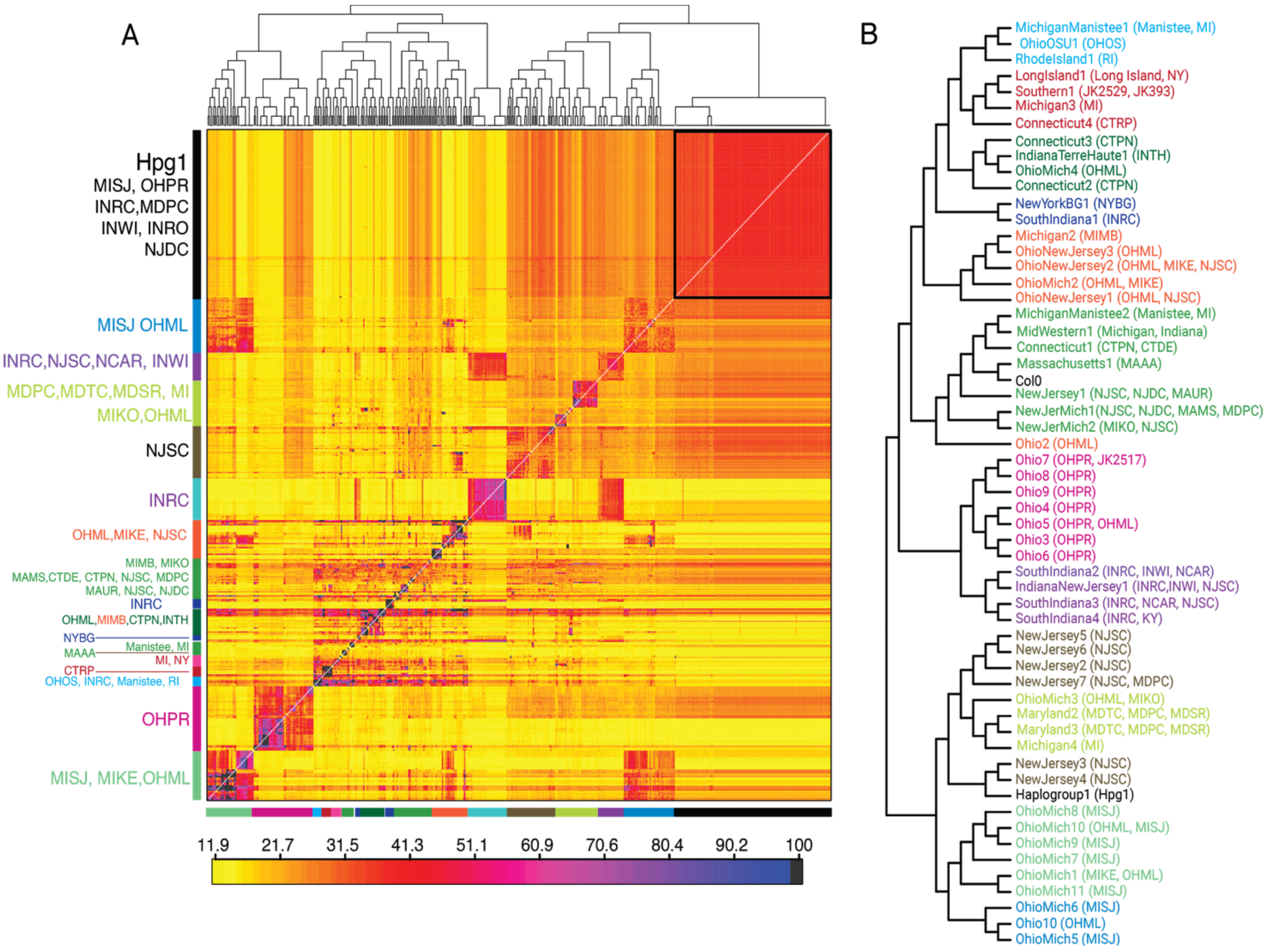

Figure S3. Identification of different haplogroups in N. American individuals

A. Co-ancestry matrix generated by CHROMOPAINTER analysis of all N. American individuals against each other, and ordered by fineSTRUCTURE analysis, **B.** Collapsed fineSTRUCTURE tree generated by merging individuals into groups (populations from which the individuals were collected are shown in parentheses, herbarium individuals are denoted by JKxxx).

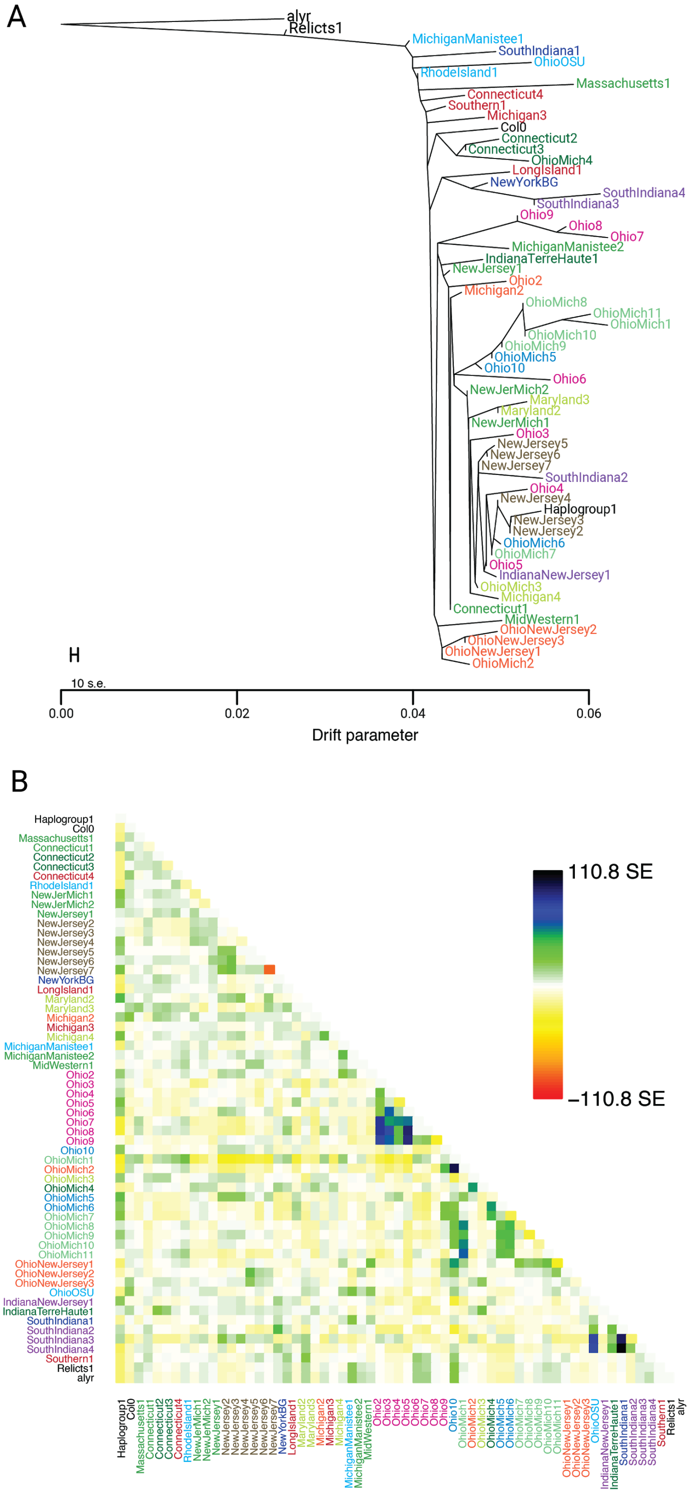

Figure S4. Maximum likelihood tree of N. American groups using Treemix

A. Maximum likelihood (ML) tree of N. American groups defined by fineSTRUCTURE with no migration edges inferred, and *A. lyrata* (*alyr*) as outgroup. **B.** Residuals of ML tree. Residual covariance between any pair of groups derived from fineSTRUCTURE clustering divided by average standard error (in pairs, hence scaled residuals). Positive residuals indicate over-estimated covariance between a pair of populations (in green to dark blue shades), which are candidates for admixture.

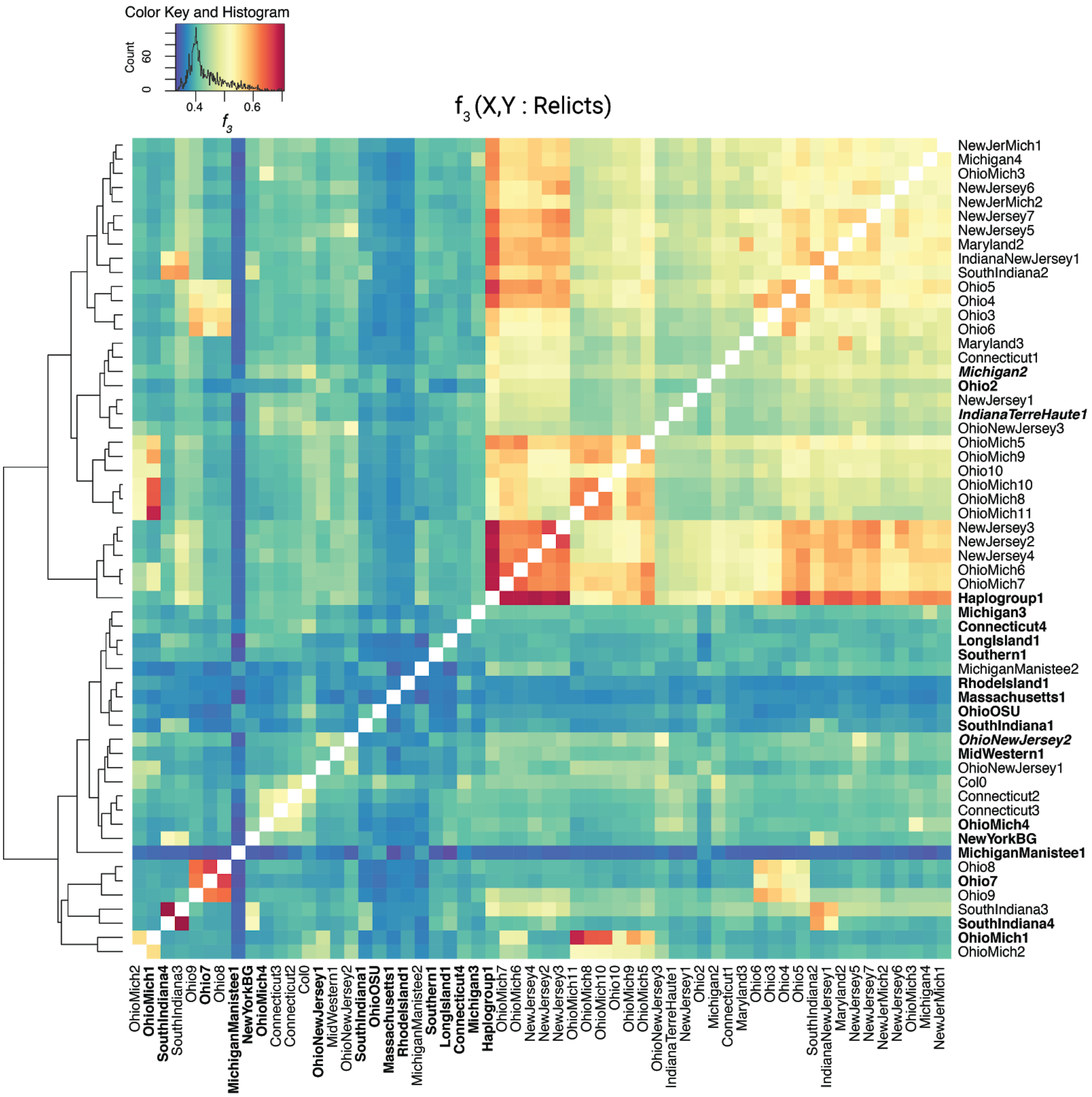

Figure S5. Shared drift among N. American haplogroups using f_3 outgroup analysis

Outgroup f_3 statistic in the form of (X,Y: relicts), where X and Y are two test N. American haplogroups. The heatmap shows the extent of shared drift among all the pairs of haplogroups (red: high shared drift; blue: low shared drift).

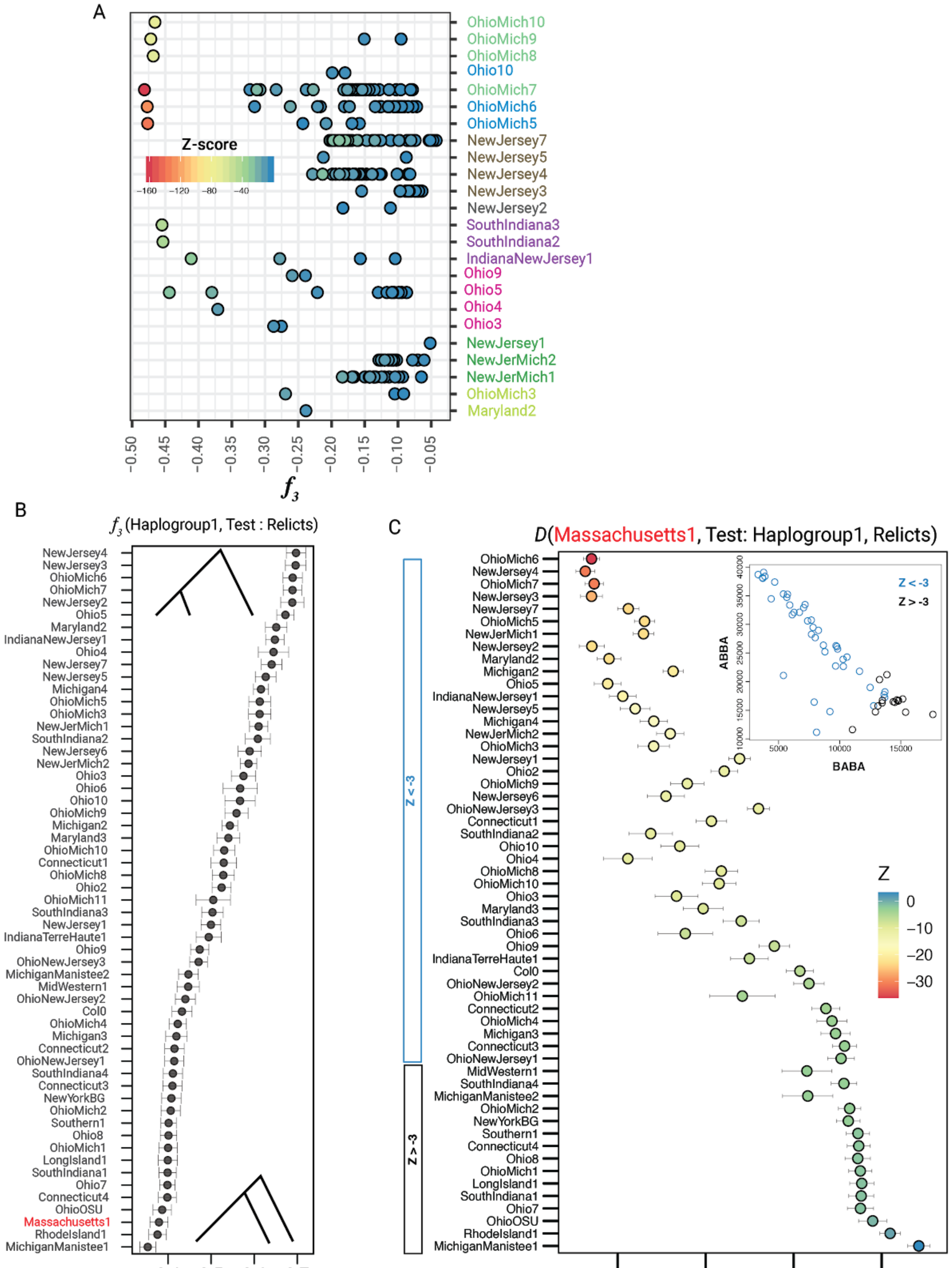

Figure S6. Admixed groups and shared drift of Haplogroup1 with other haplogroups

A. f_3-statistic for detection of admixture in groups. Tests were performed with all the $f_3(group_i, group_j : group_k)$ configurations possible, where groups *i*, *j* and *k* are three distinct N. American groups. Scores of the groups ($group_k$) with significant negative f_3 scores (Z-score < -3) are shown, with Hpg1 as either $group_i$ or $group_j$ in the test configuration. **B.** Outgroup f_3-statistic in the form of (Hpg1, test: relicts) to determine allele sharing between Haplogroup1 and other groups. **C.** *D*-statistics was then used in the form of (Massachusetts1, test: Hpg1, Relicts) to determine significant allele sharing (gene flow) between Haplogroup1 and test groups. Groups with Z-score < -3 are colored in blue and the rest in black. Inset: Count of BABA sites plotted against count of ABBA sites.

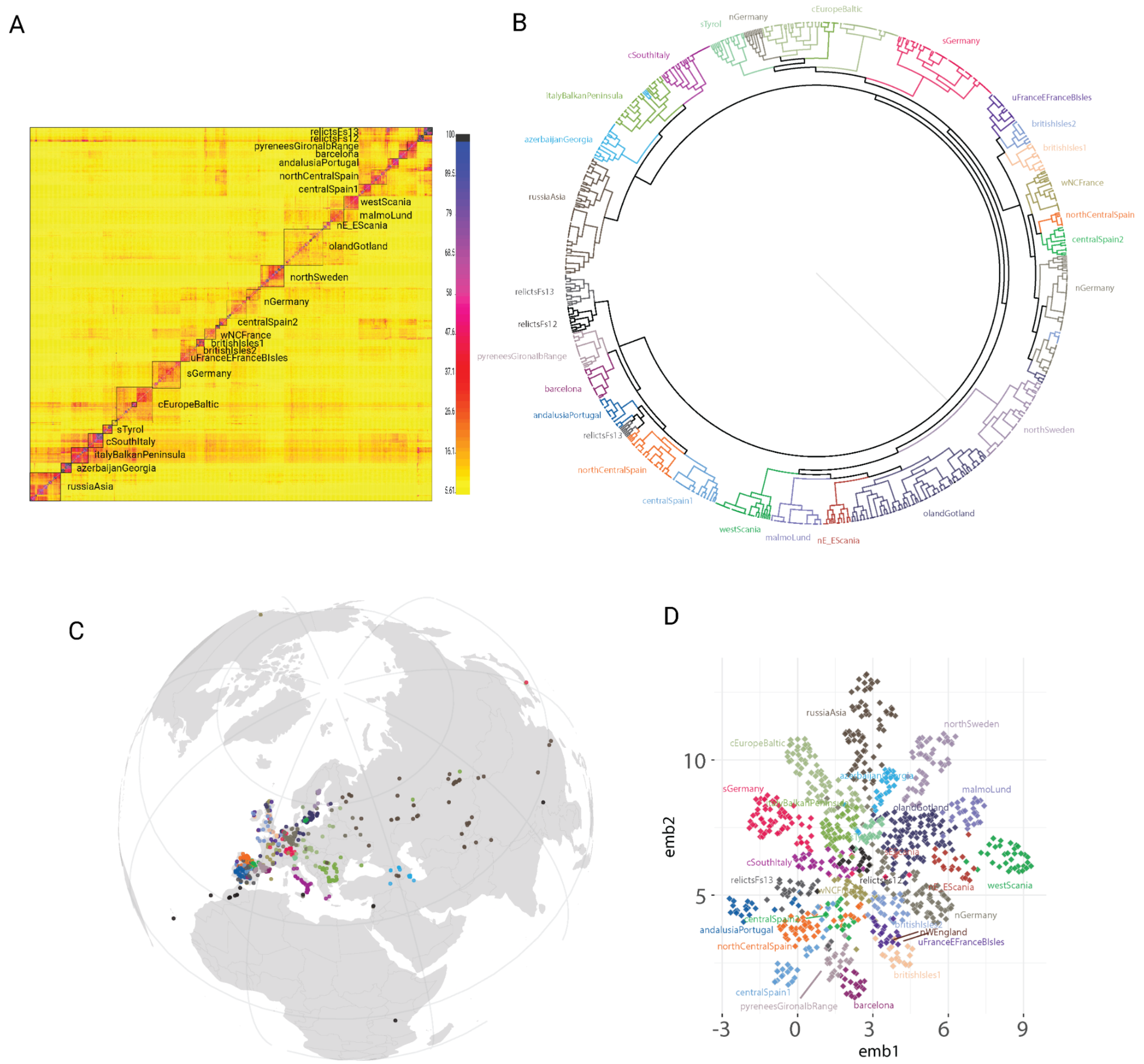

Figure S7. **fineSTRUCTURE clustering of non-American *A. thaliana* individuals from the native range and chromosome painting of N. American groups with the *regions* as donors**

A. Co-ancestry matrix of *A. thaliana* derived by chromosome painting of individuals from the 1001 Genomes project (excluding N. American individuals) and additional genomes from China, Ireland and Africa (using CHROMOPAINTER). Subsequent fineSTRUCTURE clustering resulted in 25 *regions* and 157 sub-clusters. **B**. Collapsed fineSTRUCTURE tree generated by merging individuals into *regions* **C.** Geographic locations of individuals from (A,B) colored by their membership in one of the 25 *regions*. **D.** UMAP embeddings derived from co-ancestry matrix presented in A.

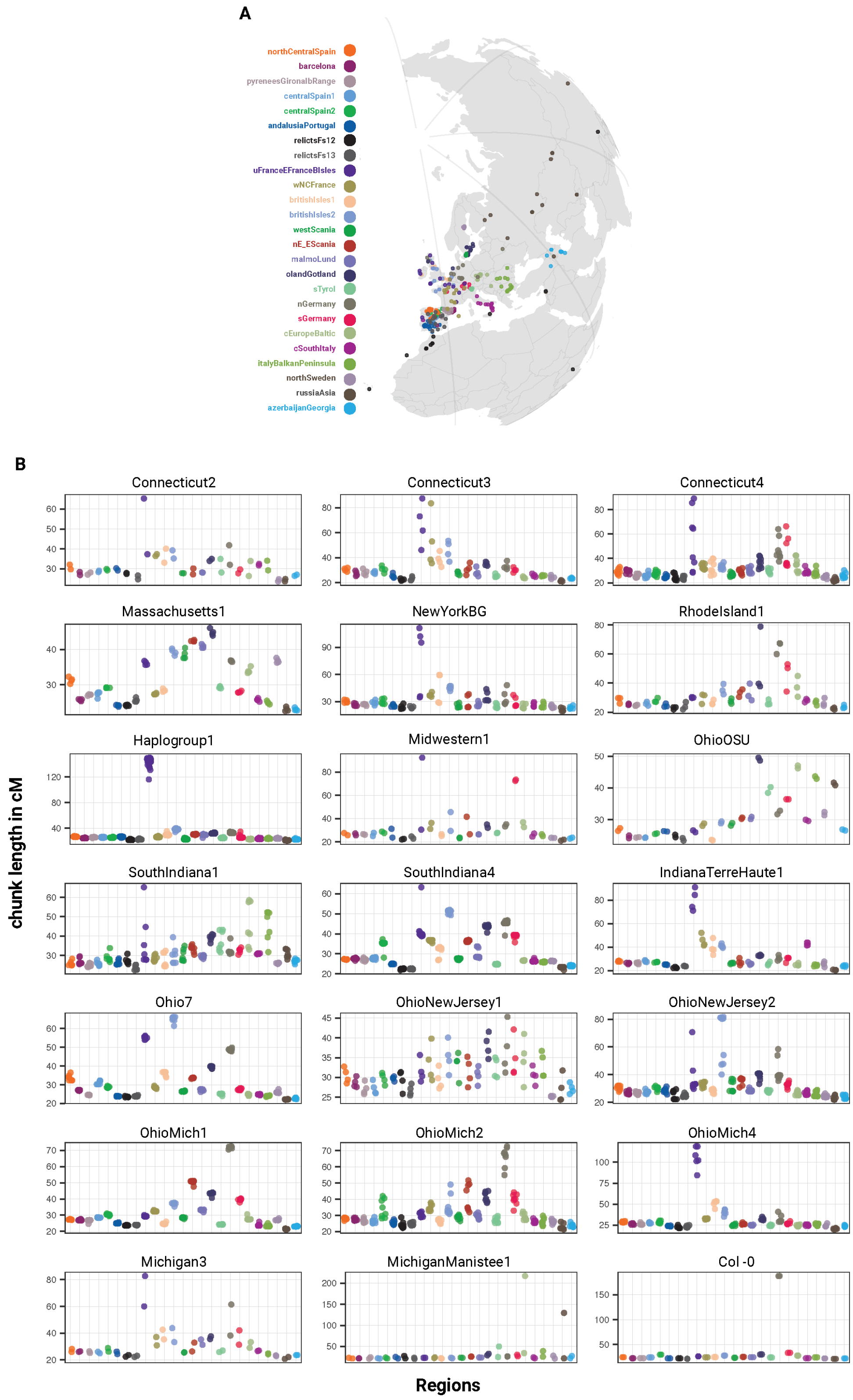

Figure S8. Haplotype copying profiles of different N. American haplogroups with AEA reference panel AEA reference panel comprising of 15 individuals per *region* was used to infer haplotype sharing patterns with N. American haplogroups (copying length on *y*-axis are in *centi*Morgans).

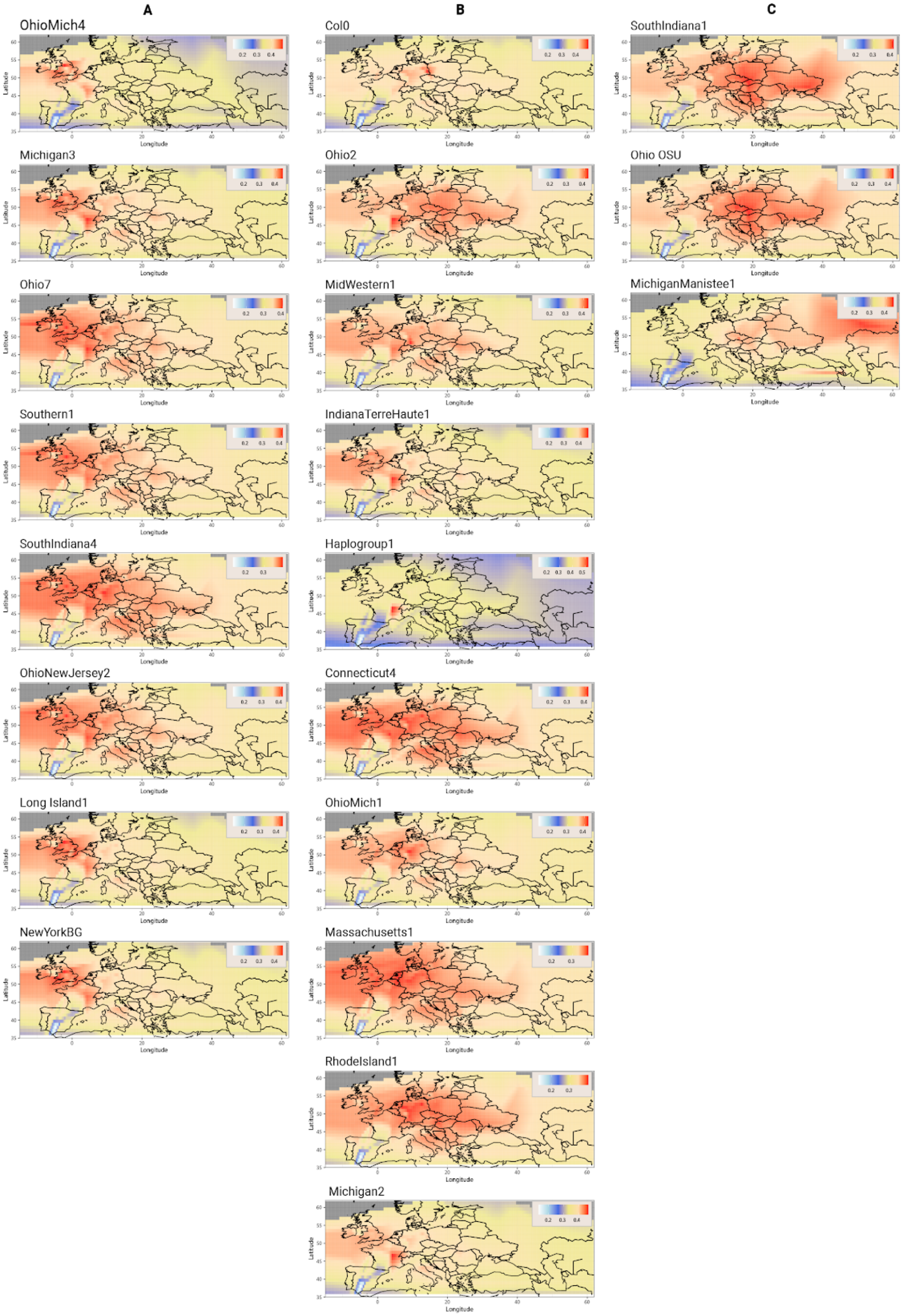

Figure S9. Shared drift of N. American haplogroups with AEA sub-clusters with f_3 outgroup analysis. N. American haplogroups with excess shared drift (as inferred from f_3-statistics) to **A.** Western Europe (mainly British Isles), **B.** Central Europe, and **C.** Eastern Europe. Legends in the upper right corners show f_3 values.

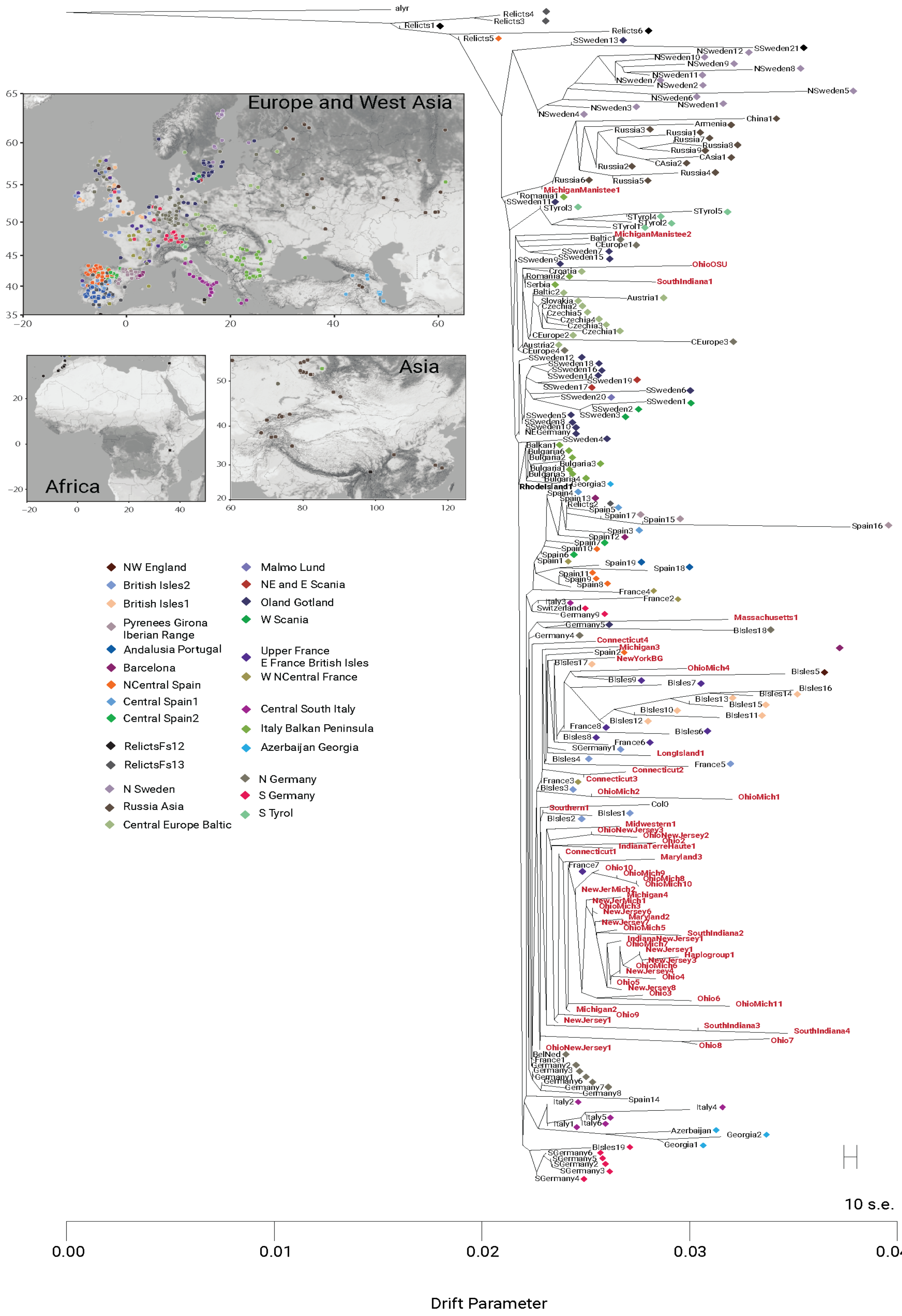

Figure S10: Maximum likelihood tree of North American groups and AEA *sub-clusters*

Maximum likelihood (ML) tree inferred using Treemix algorithm. North American *groups* (in red) and AEA *sub-clusters* (in black, and diamond colored according to that *sub-cluster*'s assignment to an AEA *region*) that

were inferred using fineSTRUCTURE were used as input and *A. lyrata* (alyr) was set as an outgroup.

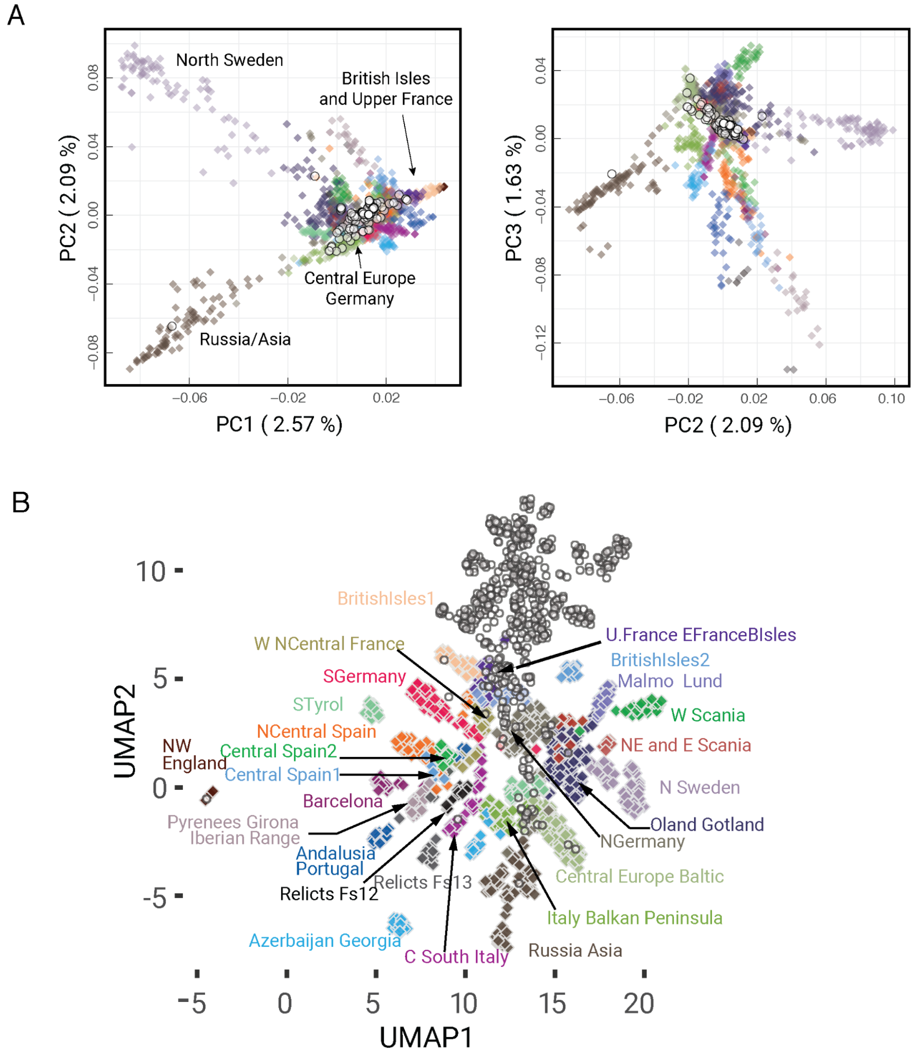

Figure S11. N. American and AEA individuals in reduced dimensions.
A. Projection of N. American individuals (white circles) in PC space derived from individuals of 27 Afro-Eurasian regional clusters. **B.** Uniform manifold approximation and projection (UMAP) embeddings of the first 50 PC components derived from PCA (without projection for N. American individuals) of all individuals. (White circles: N. American individuals.)

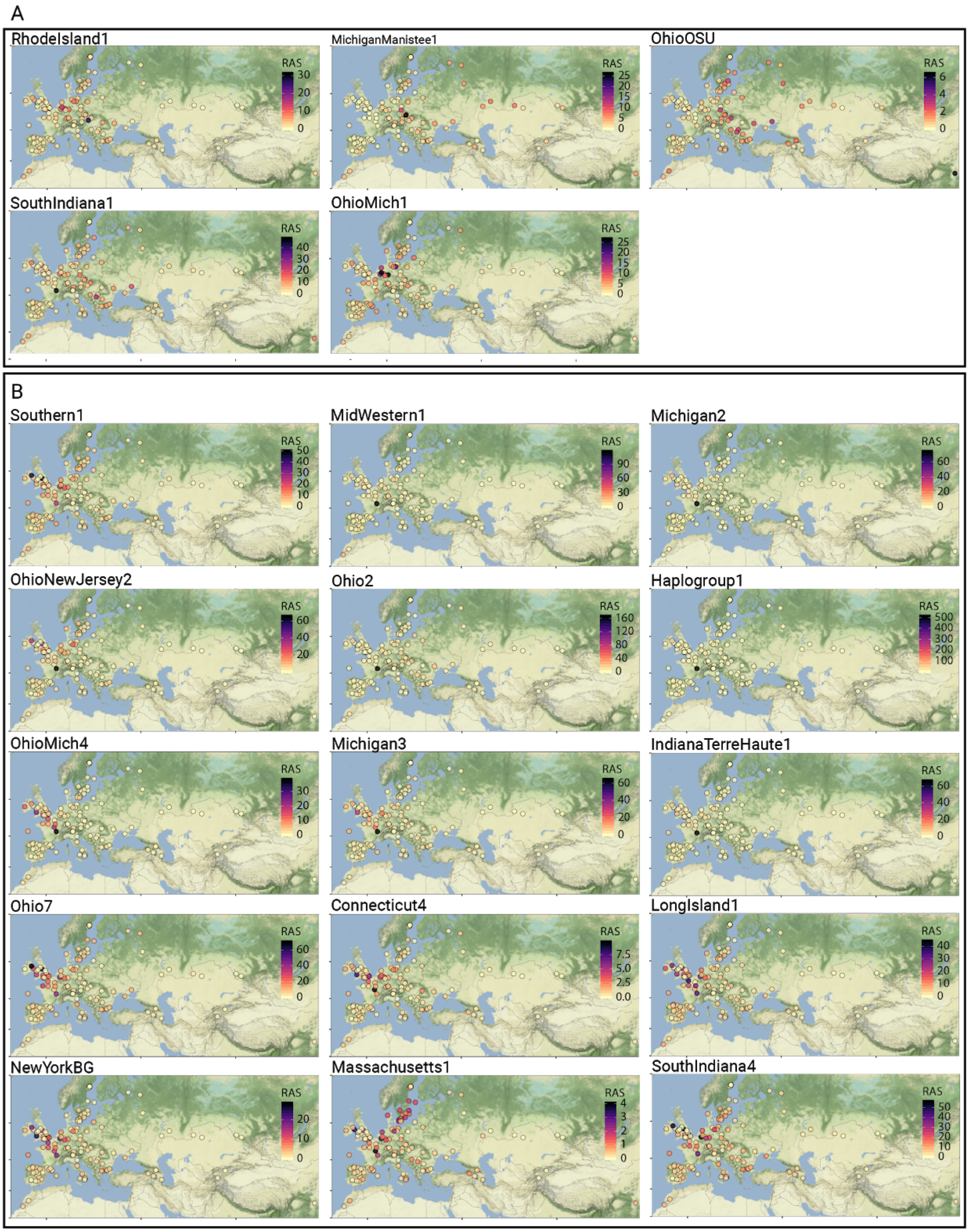

Figure S12. Rare allele sharing of N. American haplogroups with AEA sub-clusters
N. American haplogroups with excess rare allele sharing (RAS) with **A.** central/Eastern Europe, and **B.** Western Europe (mainly British Isles).

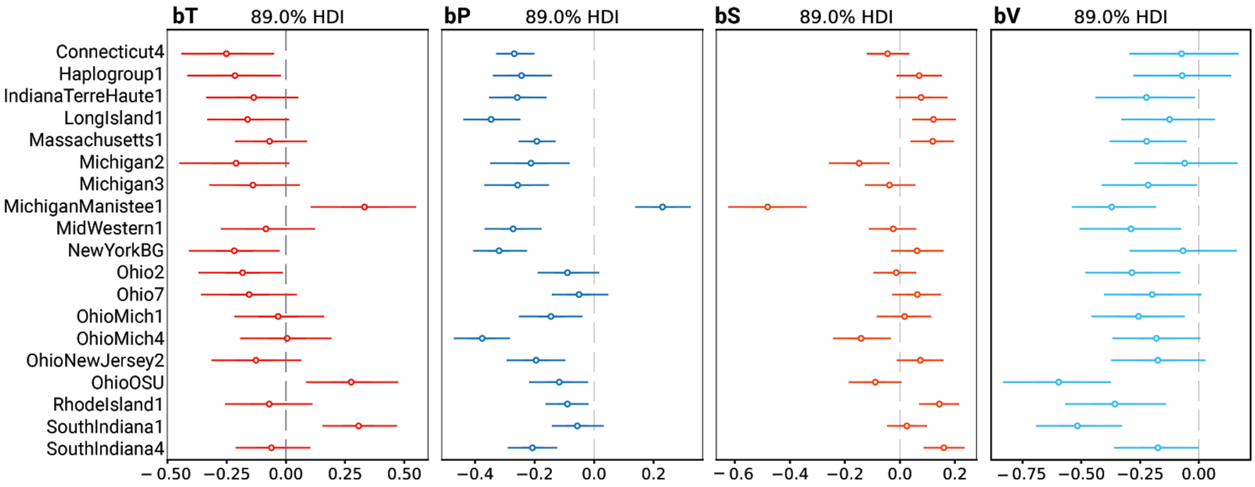

Figure S13. Posterior means and 89% compatibility interval for individual group's β coefficients tavg (bT), precipitation (bP), solar radiation (bS) and water vapor pressure (bV).

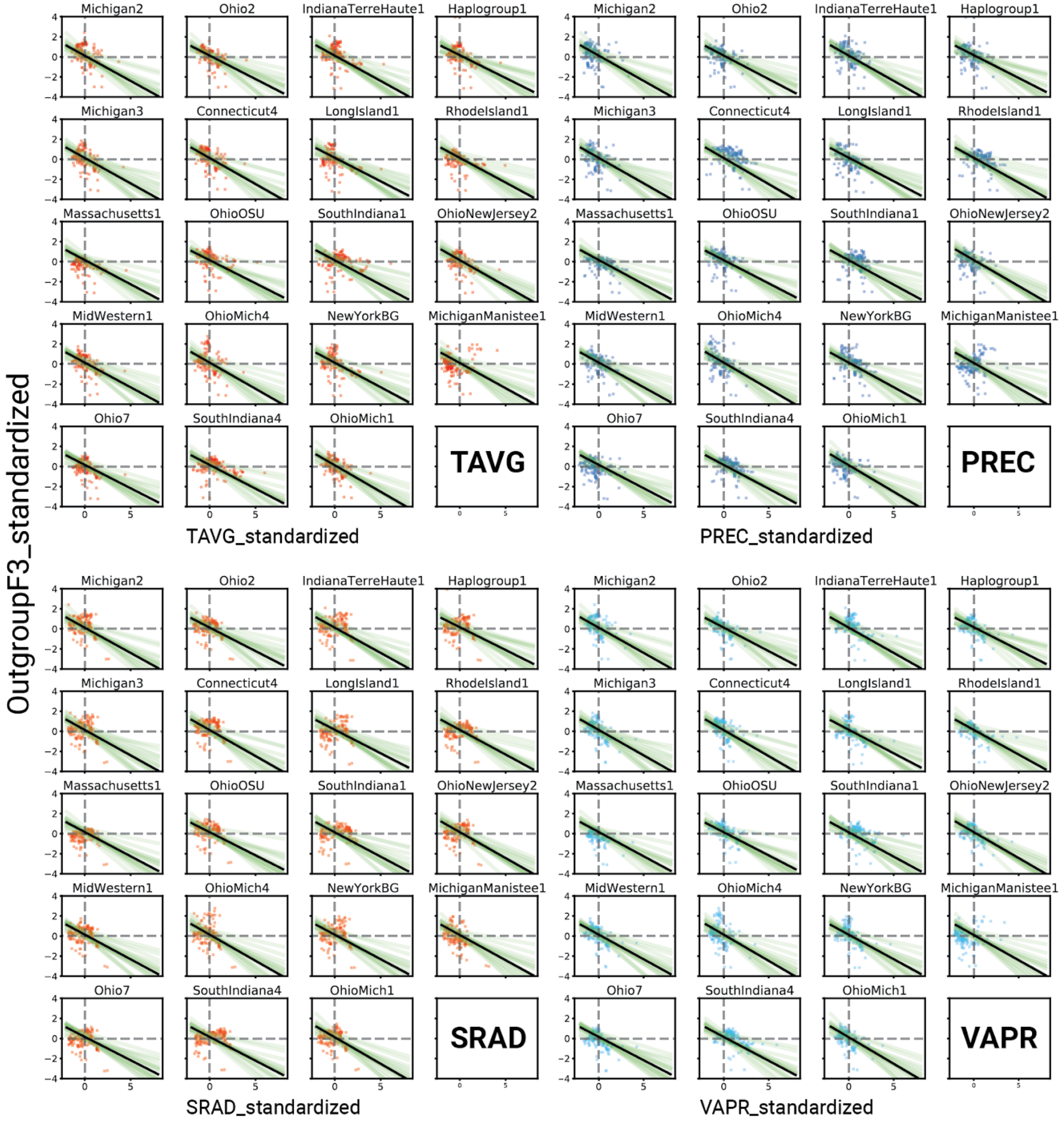

Figure S14. Sampled posterior regression lines for the model describing the relationship between *f_3*-outgroup statistics and environmental variables

For every N. American group, mean estimate (solid black line) of the posterior regression lines (thin green lines) was plotted against each environmental variable. X-axis is the standardized environmental variable and y-axis the standardized f_3 outgroup statistic derived from the configuration: N. American group, AEA-subcluster$_i$: relictsFs12 (as outgroup). The thin green regression lines show overall uncertainty for each group.

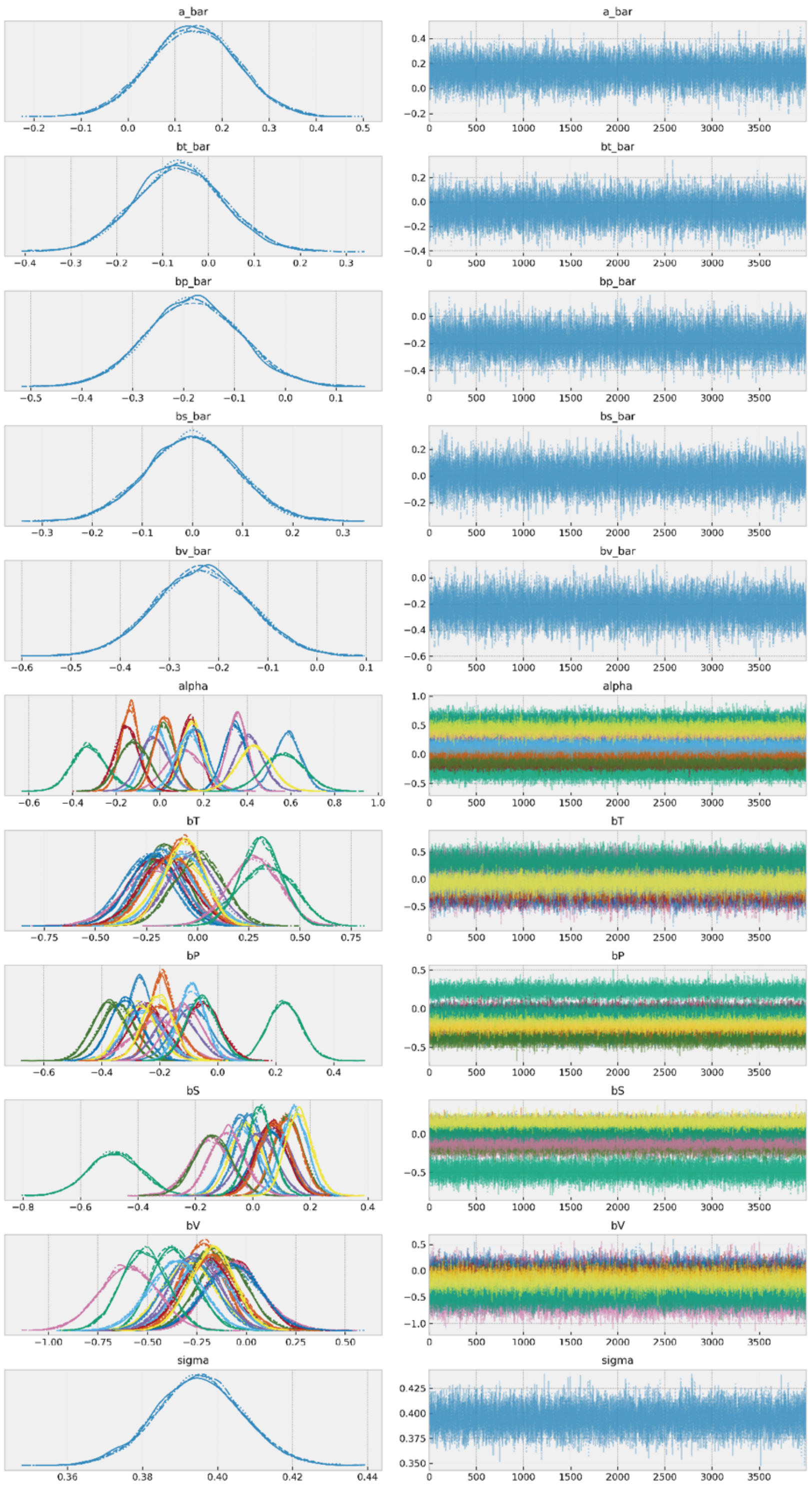

Figure S15. Trace plots for Bayesian Multi-level Model (bMLM)

Left columns are marginal values of the trace for different parameters (a_bar = pooled intercept, bt_bar = pooled β coefficient for tavg (°C), bp_bar = pooled β coefficient for precipitation (mm), bs_bar = pooled β coefficient for solar radiation (kJ m^{-2} day^{-1}) , bv_bar = β coefficient for water vapor pressure (kPa), alpha, bT, bP, bS and bT are the respective individual group's β coefficients). Right columns are the model traces from 4,000 iterations after 1,000 tuning iterations for the parameters.

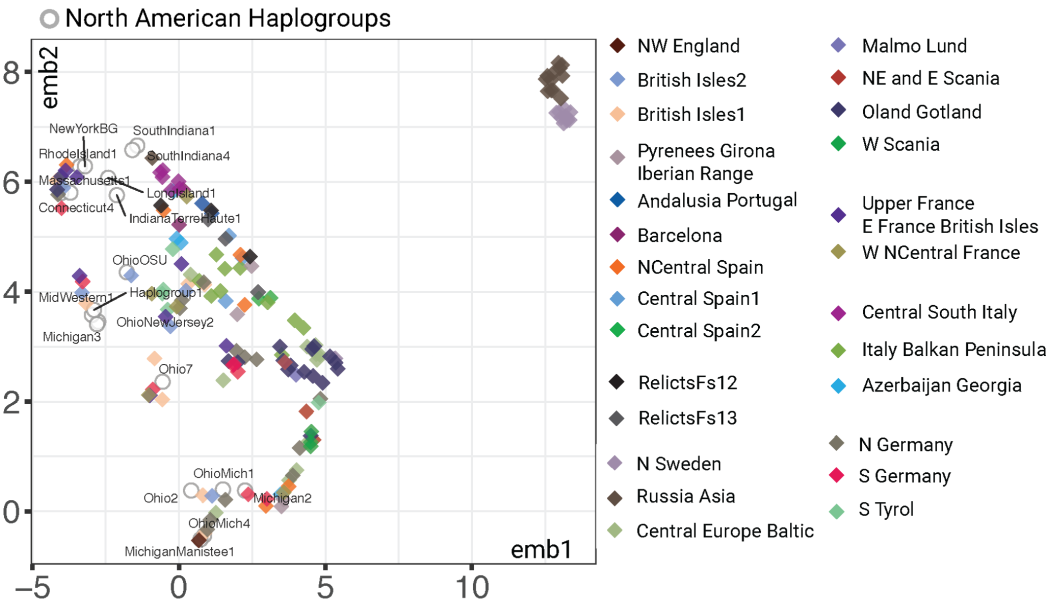

Figure S16. Projection of populations consisting of N. American *groups* and AEA *sub-clusters* in reduced environmental dimensions

UMAP embeddings showing populations in reduced dimensions of environmental variables T_{avg} (°C), precipitation (mm), and water vapor pressure (kPa).

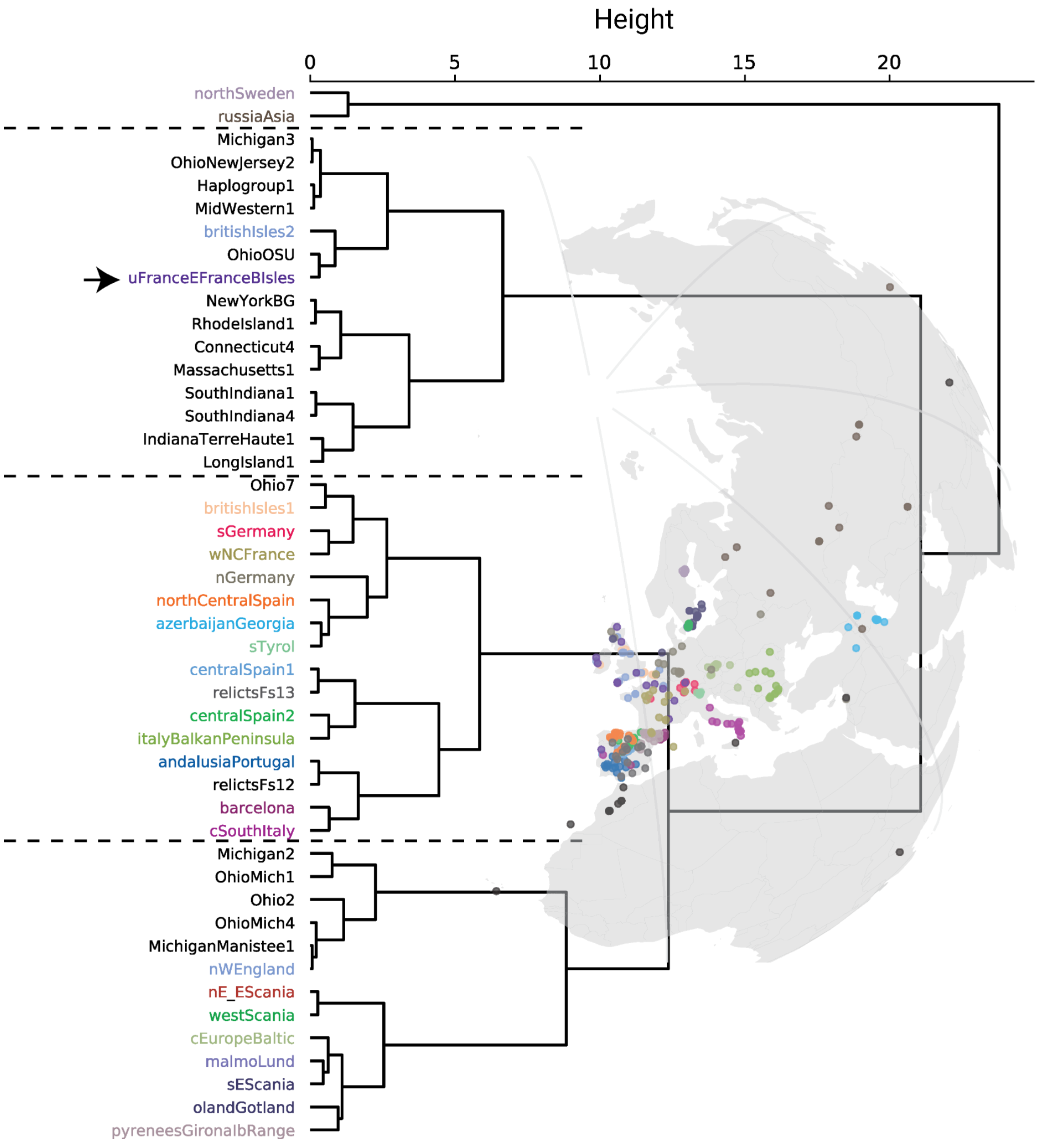

Figure S17 . Hierarchical clustering on reduced environmental space of N. American *groups* and AEA *regions*

Ward's linkage function with Euclidean distance was used on UMAP embeddings derived from environmental variables associated with AEA *regions* and N. American *groups*. (AEA individuals and their *regions* are dots on the Earth in corresponding colors on the dendrogram, N. American *groups* are in black color on the dendrogram). Dashed lines separate 4 distinct clades in the dendrogram.

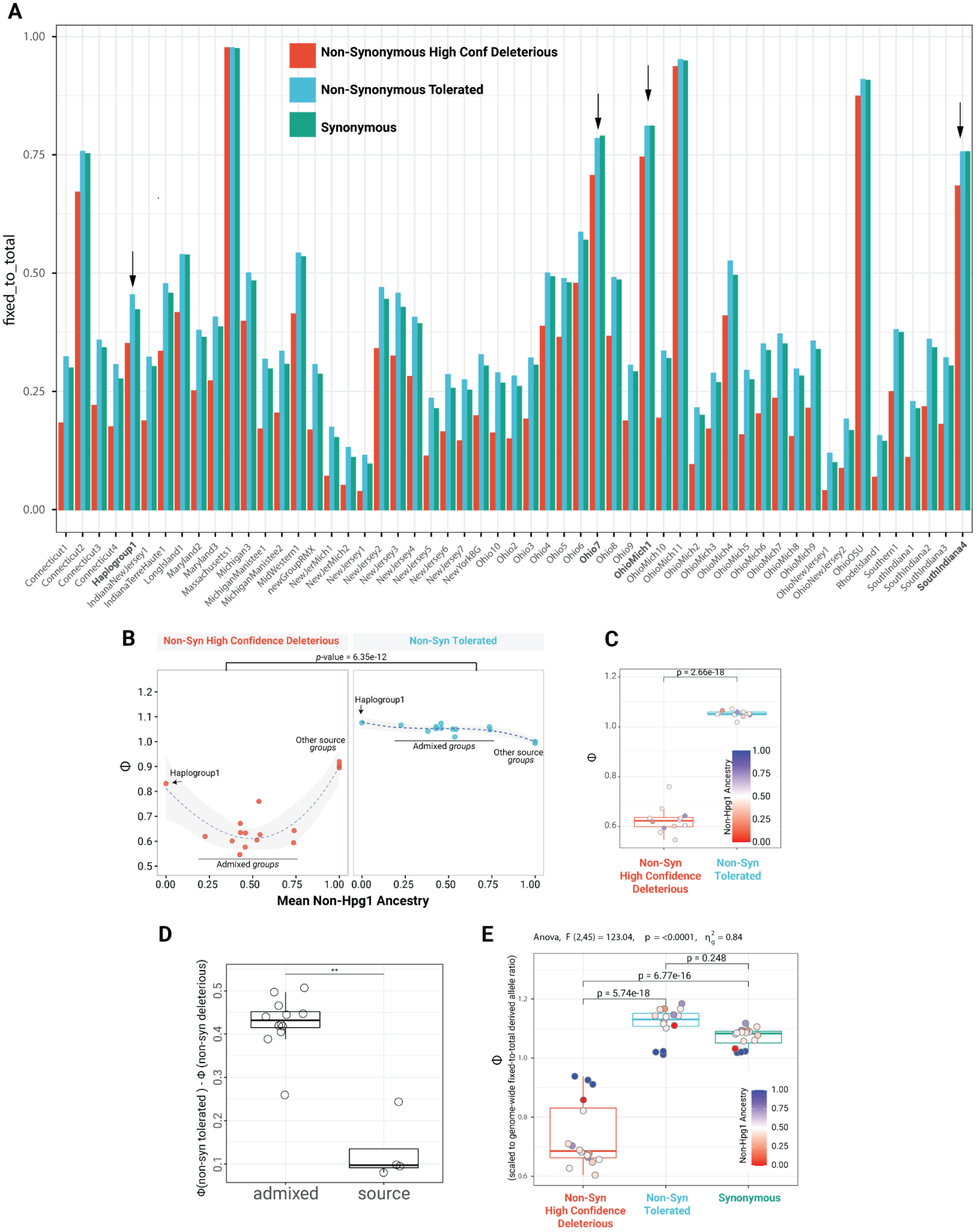

Figure S18 : Scaled fixed-to- total derived alleles ratio for the N. American *groups*

A. Raw fixed-to-total derived alleles ratio across the N. American groups. Derived alleles were separated in three categories: synonymous, non-synonymous tolerated (SIFT 4G score > 0.05), and non-synonymous high

confidence deleterious (SIFT4G score < 0.05). Arrows point to the *groups* that are sources of admixture in our focal populations (OHPR, MISJ, INRC and NJSC) . **B**. Scaled fixed-to-total derived alleles ratio (Φ, using only synonymous mutations) in different categories in the *groups* of focal populations against mean non-Hpg1 ancestry. (dotted curve: polynomial regression curve of third degree). **C.** Φ (scaled with synonymous mutations) for only admixed *groups* (Welch's *t*-test *p*-value= 2.66e-18). **D.** Comparison among the admixed and source *groups* for the change in the Φ (non-synonymous tolerated) to Φ non-synonymous deleterious (Welch's *t*-test, p-value = 0.00147). **E.** Φ estimated by scaling to genome-wide fixed-to-total derived alleles ratio in the three categories. Means were compared with Welch's *t*-test (*p*-values as horizontal bars). ANOVA was performed to compare differences among means of all the categories.(dark red: source group Hpg1, dark blue: other source groups)

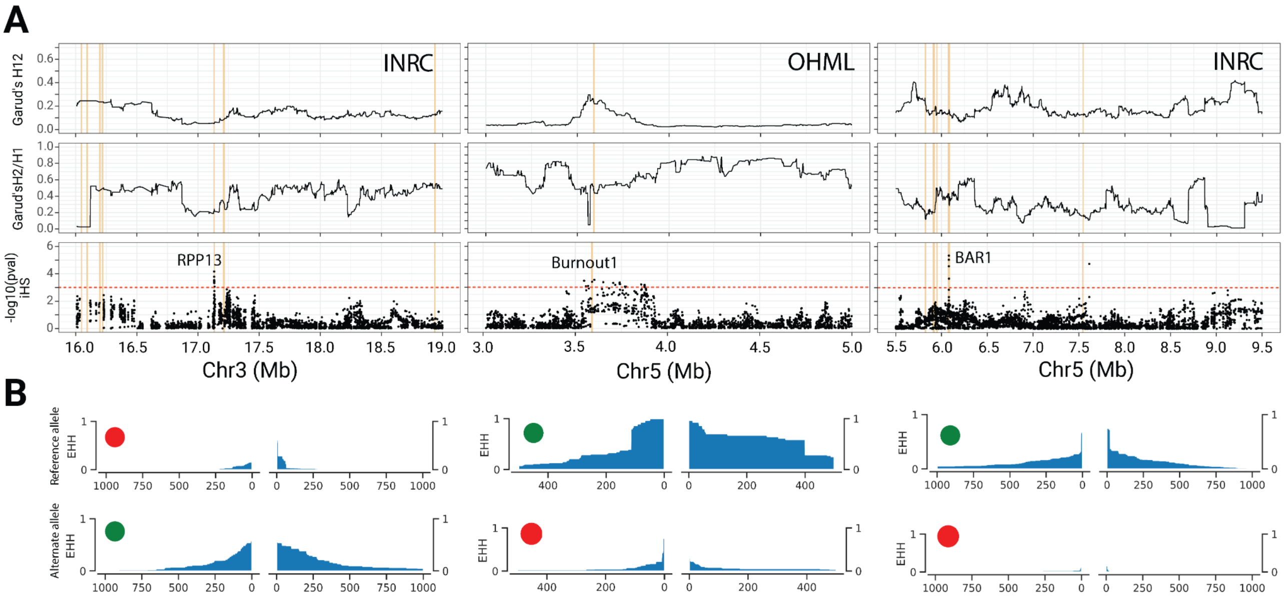

Figure S19: Haplotype homozygosity based statistics on three NBS-LRR domain containing genes
A. Garud's H_{12} and H_2/H_1 statistics and |iHS| scores around three disease resistance/NLR genes. **B**. Decay of extended haplotype homozygosity (EHH) around the variants with the lowest |iHS| *p*-value in the three genes shown in (B). Green dot indicates allele with selected variant, and red dot alternative allele. X-axis gives distance from the focal variant as the number of flanking variants in the data set.

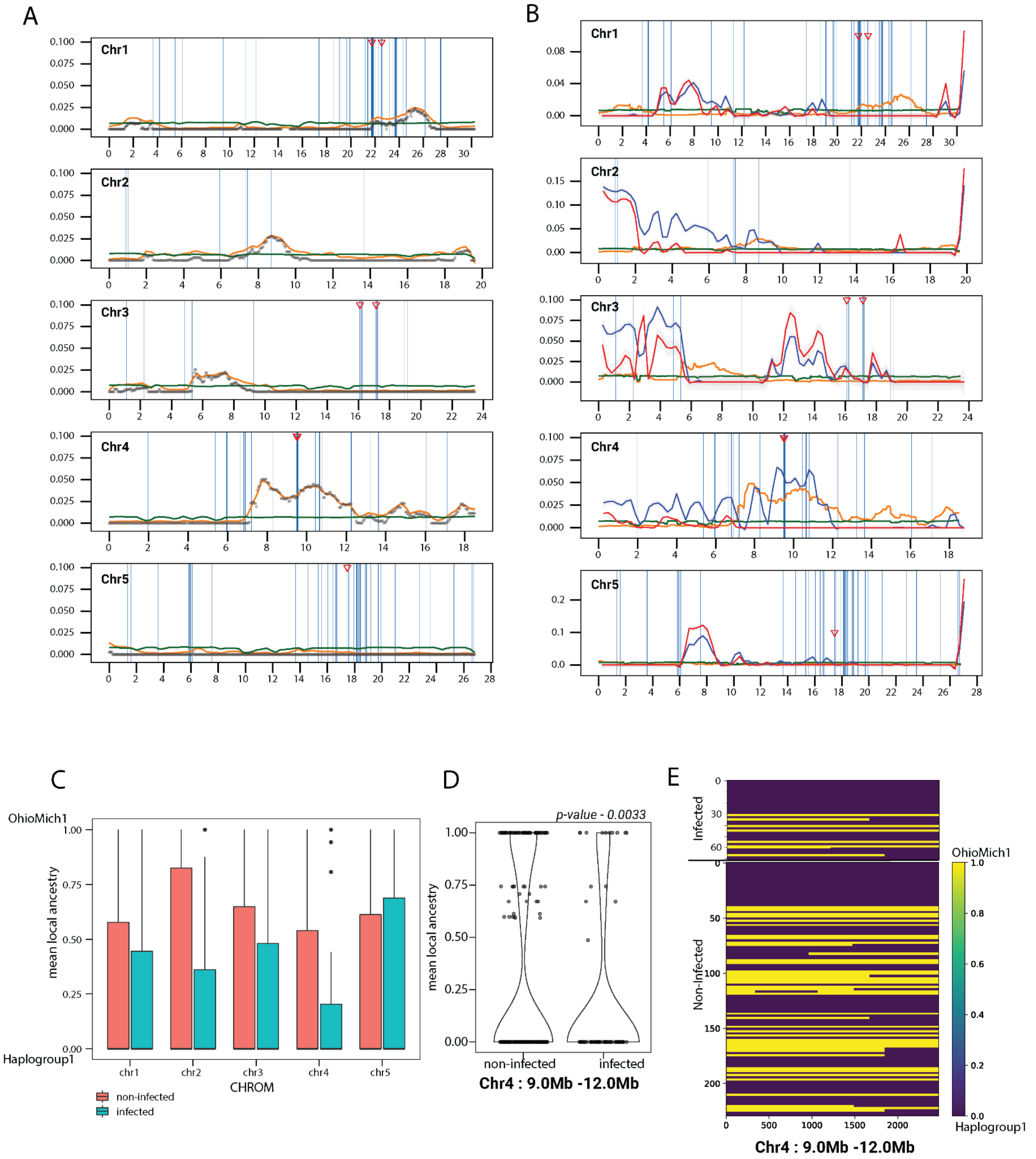

Figure S20. Differentiation between infected and non-infected individuals of population MISJ
A. Fst values (*y*-axes) across 5 chromosomes between infected and non-infected individuals of the population MISJ (black dots), random assignment of individuals to the two groups (over 1000 permutations) results in the differentiation level of the green line. Orange line represents sub-sampling of both the groups to ⅔ individuals (1000 permutations) **B.** Blue line represents differentiation of non-infected individuals to Hpg1 and red line shows differentiation of infected individuals to Hpg1 across 5 chromosomesGreen and red lines are same as described for A **C.** Distribution of mean local ancestry in infected (blue) and non-infected

individuals (red). **D.** Mean local ancestry on Chromosome4: 9Mbp-12Mbp where RPP4/5 resistance gene cluster is located (*p*-value = 0.0033) between two different pools of plants. **E.** Local ancestry block in the region containing RPP4/5 (purple: Hpg1, yellow: OhioMich1).

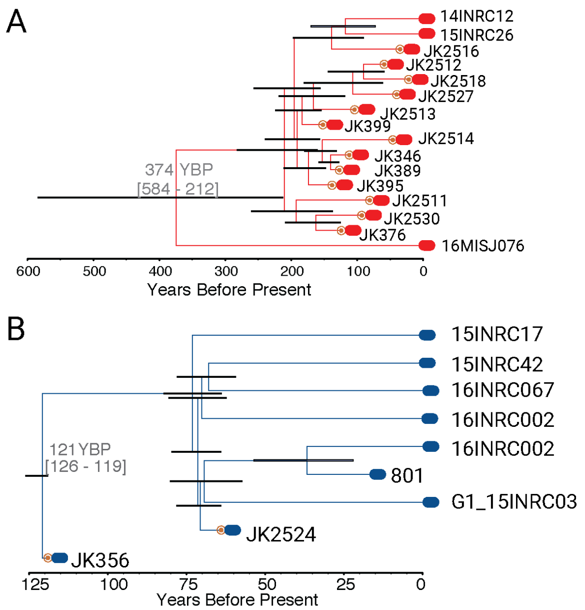

Figure S21. Maximum Clade Credibility (MCC) trees
A. *group* Hpg1, **B.** *group* SouthIndiana4.

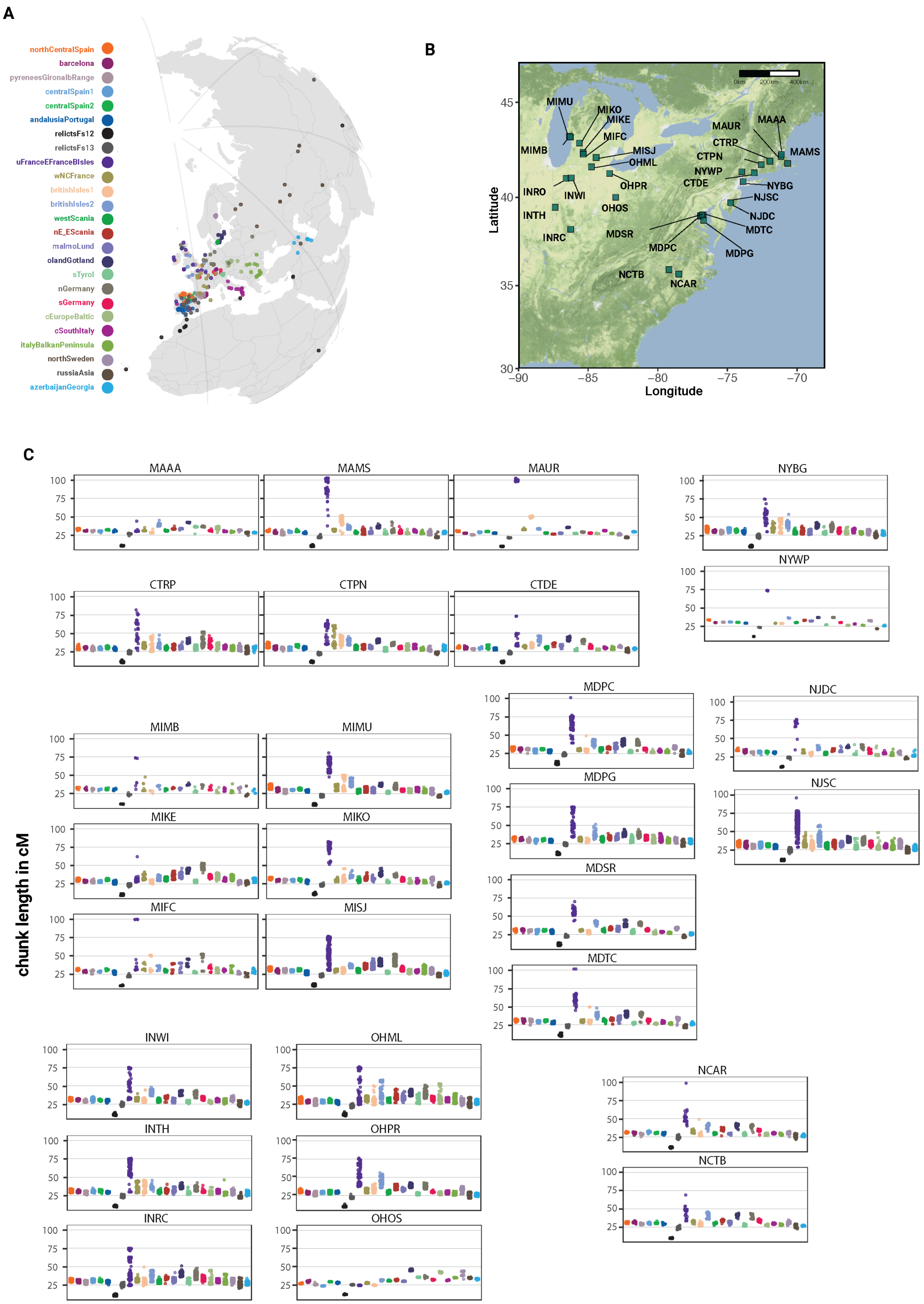

Figure S22. Population wide haplotype sharing of RAD-seq genotyped North American individuals
A. AEA regions and geographic locations of the representative individuals from these regions used as a reference set for chromosome painting of N. American populations, **B.** Geographic locations of N. American populations, **C.** Haplotype sharing of individuals of N. American populations (total genomic chunks copied from a given AEA region expressed in cM)

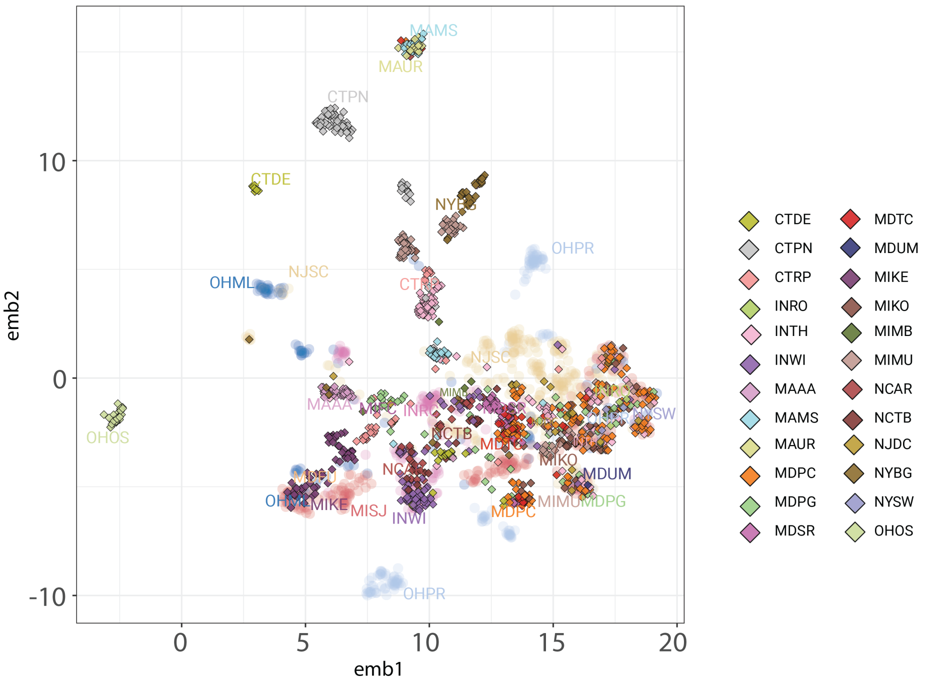

Figure S23. Genetic variation in the RAD-seq genotyped individuals

UMAP embeddings of compositional ancestry matrix of RAD-seq genotyped individuals painted with a reference panel of AEA regions. Fuzzy round shapes represent populations that had more individuals sequenced with WGS than the populations in the diamond shape.

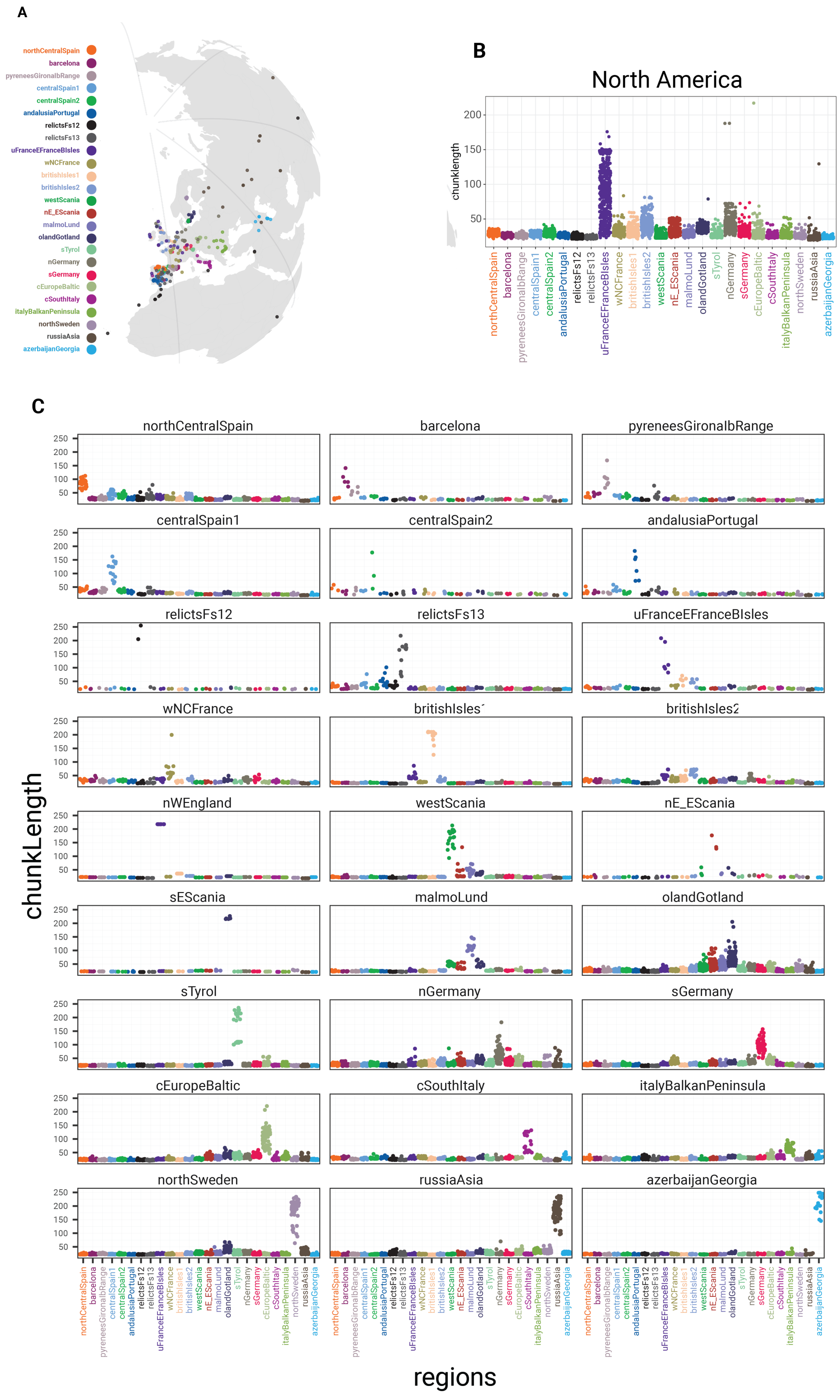

Figure S24. Ancestry profiles of AEA *regions* subset individuals

A. Reference panel individuals of AEA *regions,* B,C. Haplotype copying profile of N. American *A. thaliana* individuals and individuals from every AEA *region* (chunk length is expressed in *centiMorgans*)

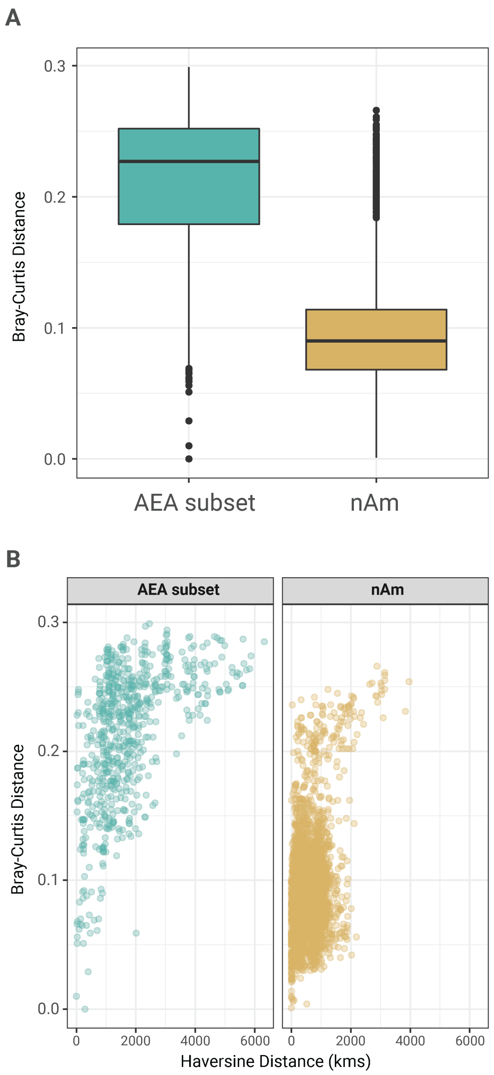

Figure S25. Compositional dissimilarity in AEA and N. American individuals

A. Distribution of Bray-Curtis distance measured in AEA "regions" (subset of AEA individuals) and N. American groups derived from ancestry profiles inferred using reference AEA panel (exclusive of the AEA subset), B. Comparison of increase in compositional dissimilarity with geographical distance among different AEA *regions* and N. American *groups*.

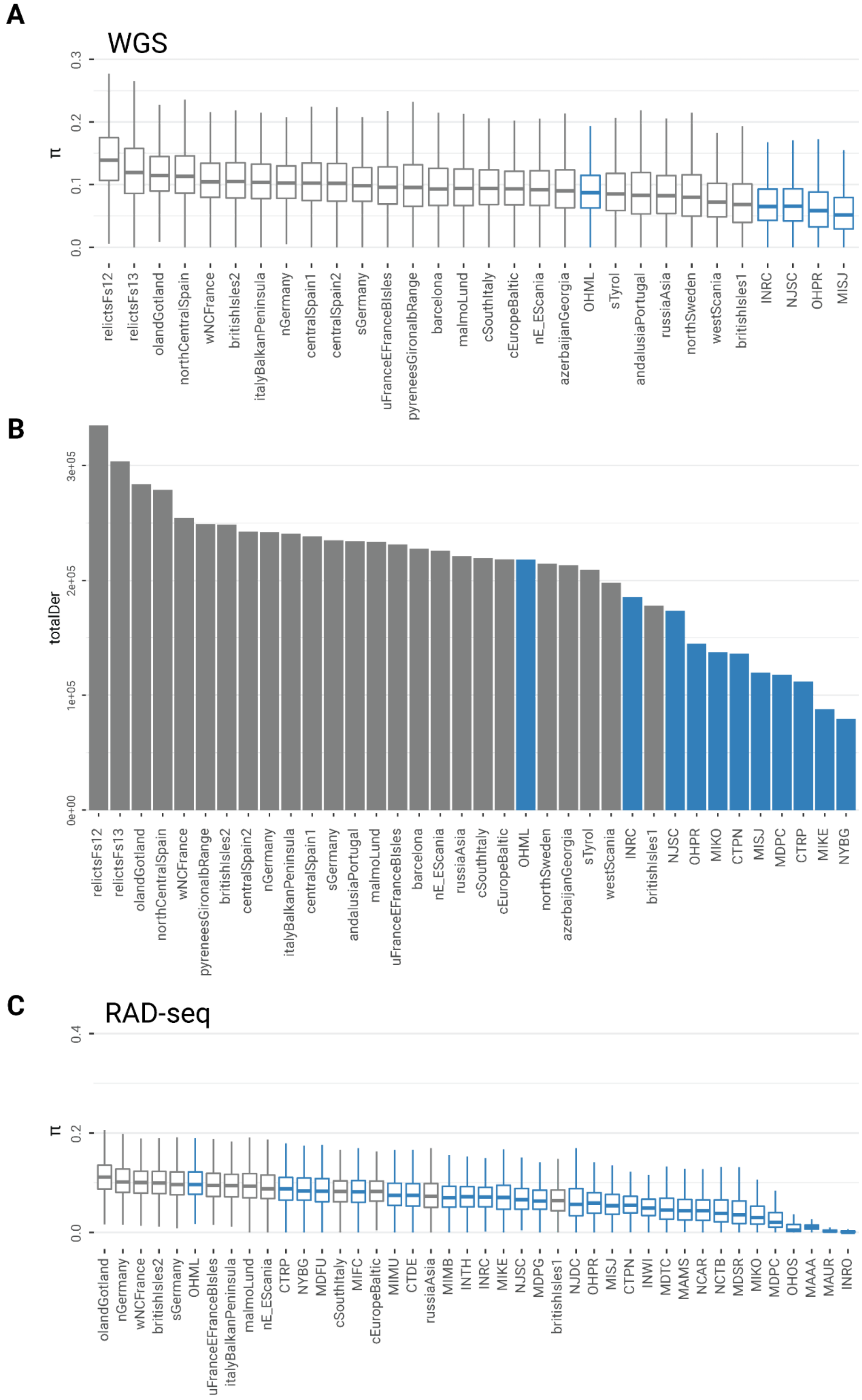

Figure S26. Measure of diversity in AEA *regions* and N.American populations

A. Nucleotide diversity (π) measured in AEA *regions* (gray) and focal N. American populations(blue) with WGS data (window size = 50,000 bp, step size = 5000 bp), **B**. Derived allele count in AEA *regions* and N. American populations (calculated with 10 randomly chosen individuals per *region*/population) using WGS data. **C.** π measured (window size = 500,000 bp, step size = 50,000 bp) in select AEA *regions* (gray) and all N. American populations(blue) with RAD-seq data.

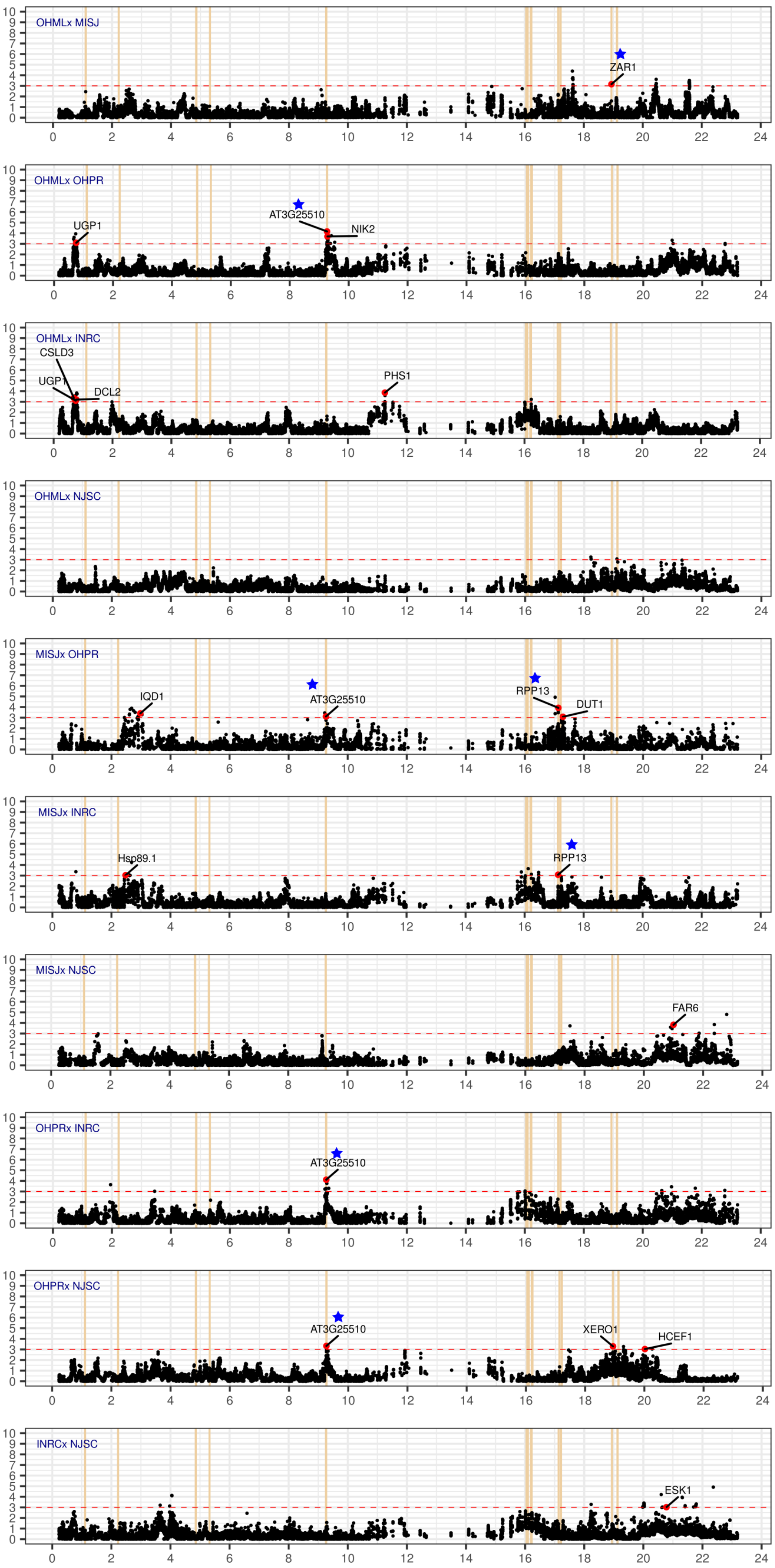

Figure S27. p-values for xpEHH (cross-population extended haplotype homozygosity) scores among all population comparisons Blue stars are genes encoding proteins with NBS-LRR domains (immune system genes). *y*-axis is -log10(p-value)) on chromosome 3.

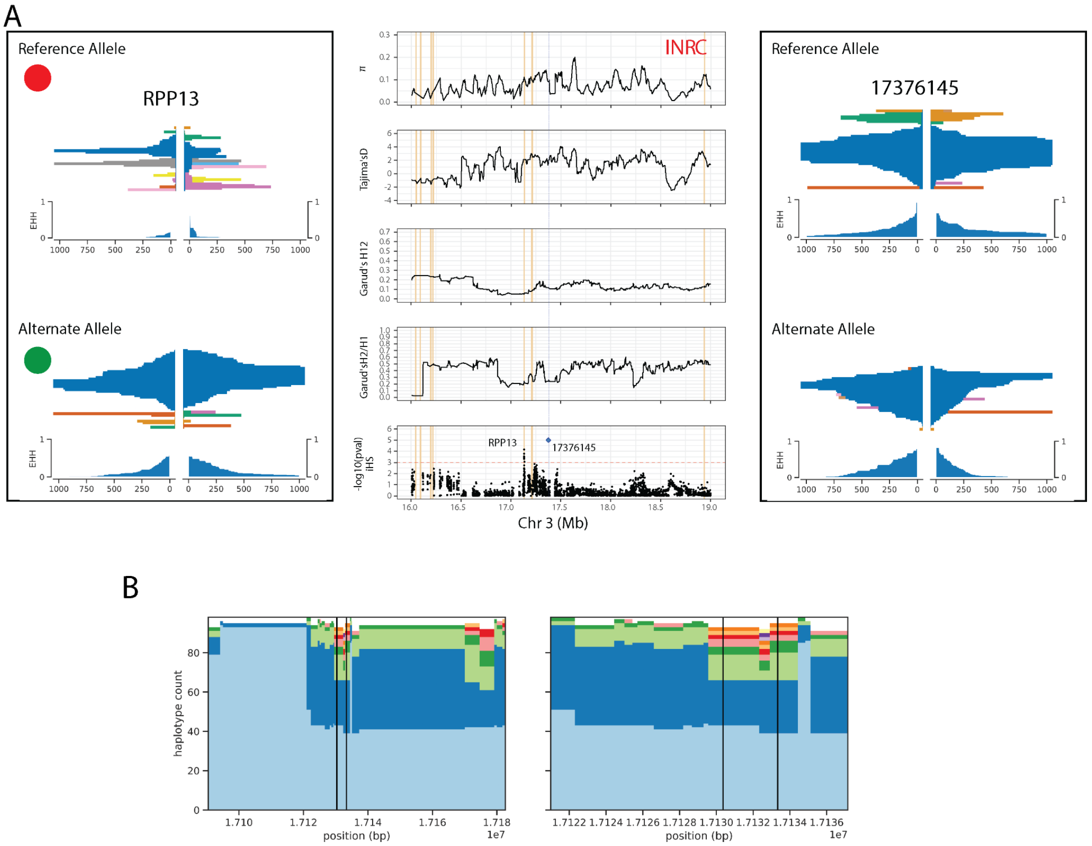

Figure S28. Comparison of selected SNP's haplotype homozygosity block with a nearby SNP with similar allele frequency

A.Left column shows haplotype plots and decay of haplotype homozygosity on the reference and alternate allele *RPP13*. The middle section shows different statistics (***π***, Tajima's D, Garud's H12 and H2/H1 and p-values of |*iHS*|) in genomic regions on chromosomes 3. Right column shows haplotype plots and decay of haplotype homozygosity on a nearby SNP (position noted) with same allele frequency (as selected SNPs in the left columns, respectively) but with non-significant |*iHS*| scores (EHH decay x-axis is the number of SNPs on the left and right side of the focal SNP). **B**. Haplotype frequencies in the genomic neighborhood of *RPP13* (gene is marked with the black vertical boundaries).

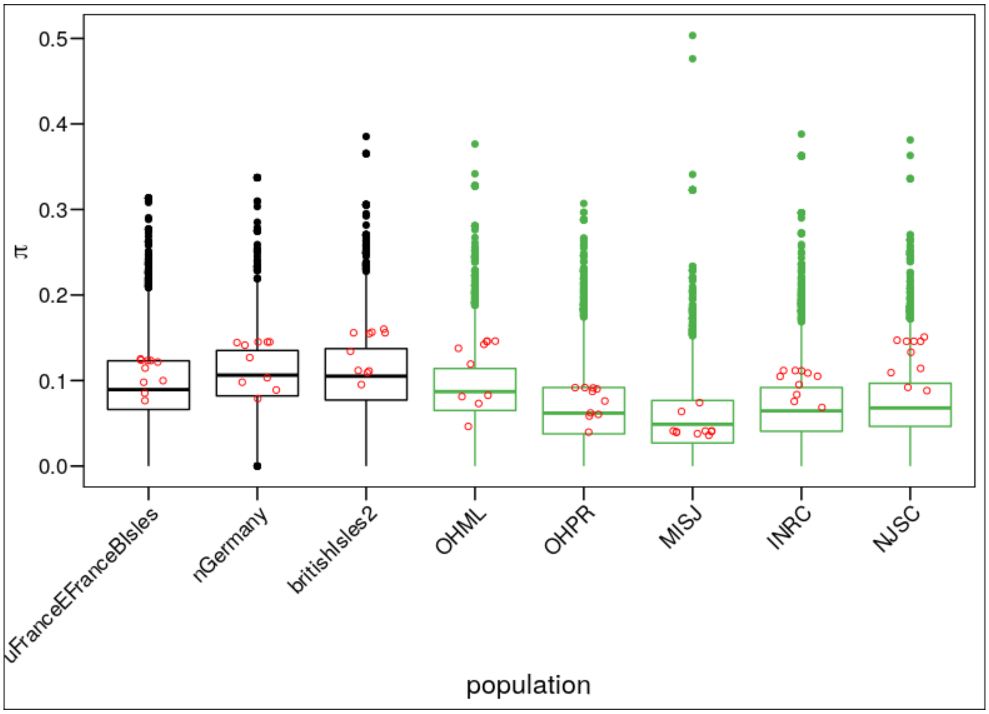

Figure S29. Nucleotide diversity (π) on chromosome 3 in AEA *clusters* and N. American populations
Boxplots in black are AEA *clusters* that contribute significant ancestry to the focal N. American *groups* observed in the populations OHML, OHPR, MISJ, INRC and NJSC (green). π was calculated in windows of 50,000 bp and step size of 5,000 bp. Red circles are π in a genomic window (17.1 Mb -17.2 Mb) that contains *RPP13*.
