# Supplementary material for: Multiple Sources of Introduction of North American Arabidopsis thaliana from across Eurasia
Source: Mol Biol Evol. 2021 Sep 9;38(12):5328–44. doi: 10.1093/molbev/msab268 (PMC8662644; doi:10.1093/molbev/msab268)
Supplement: msab268_Supplementary_Data [file msab268_supplementary_data.zip › Table S10_iHS_GO_analysisResult.pdf]

Table S10: GO term enrichment analysis for $|\mathrm{iHS}|$ significant SNPs (p-value less than 0.001)

GO_acc	Term	query item	bgitem	pvalue	FDR	Geneid
**GO:0006950**	**response to stress**	**57**	**3506**	**2.9E-05**	**0.0017**	**AT3G23010 , AT1G51480 , AT1G70700 , AT1G01680 , AT2G24150 , AT5G46450 , AT4G03460 , AT2G32680 , AT1G11530 , AT4G36690 , AT4G32551 , AT1G21750 , AT2G33050 , AT4G23240 , AT1G52330 , AT4G37310 , AT4G23170 , AT4G29770 , AT3G22690 , AT1G04120 , AT1G56540 , AT4G23140 , AT5G18360 , AT5G65970 , AT1G18910 , AT4G03520 , AT3G09260 , AT3G56700 , AT4G03260 , AT5G11250 , AT4G32960 , AT4G05420 , AT2G33700 , AT2G14080 , AT1G32960 , AT5G13220 , AT1G31470 , AT1G78000 , AT5G11630 , AT4G03510 , AT5G41550 , AT3G46530 , AT2G43480 , AT5G12000 , AT2G43510 , AT2G33060 , AT4G32970 , AT2G43535 , AT1G76930 , AT5G38340 , AT3G29320 , AT5G11200 , AT4G23210 , AT1G17880 , AT2G27020 , AT5G11150 , AT4G03470**
**GO:0050896**	**response to stimulus**	**82**	**6250**	**0.00066**	**0.04**	**AT1G18890 , AT2G43535 , AT3G23010 , AT1G25390 , AT1G59610 , AT4G00710 , AT1G51480 , AT1G78000 , AT4G10320 , AT1G70700 , AT1G01680 , AT2G24150 , AT1G17880 , AT5G46450 , AT4G03460 , AT3G47390 , AT2G32680 , AT5G63950 , AT1G11530 , AT4G36690 , AT4G32551 , AT5G50760 , AT1G21750 , AT3G59060 , AT1G19320 , AT4G23240 , AT2G24130 , AT1G52330 , AT4G37310 , AT4G23170 , AT4G29770 , AT3G22690 , AT4G22540 , AT1G25490 , AT1G04120 , AT1G56540 , AT4G23140 , AT5G18360 , AT5G65970 , AT1G18910 , AT4G03520 , AT5G65750 , AT3G56700 , AT4G03260 , AT1G48090 , AT5G11250 , AT4G32960 , AT4G05420 , AT2G33700 , AT2G14080 , AT2G33050 , AT2G39980 , AT5G13220 , AT1G31470 , AT4G04460 , AT5G11630 , AT4G03510 , AT5G41550 , AT5G51810 , AT5G59920 , AT3G46530 , AT2G43480 , AT5G12000 , AT2G43510 , AT3G57230 , AT4G32970 , AT2G33060 , AT4G03390 , AT1G32960 , AT3G09260 , AT5G38340 , AT2G29630 , AT3G29320 , AT5G48410 , AT5G11200 , AT1G76930 , AT2G27060 , AT4G23210 , AT1G24120 , AT2G27020 , AT5G11150 , AT4G03470**
GO:0007154	cell communication	36	2223	0.0011	0.068	AT1G18890 , AT4G03460 , AT4G00710 , AT1G51480 , AT1G48090 , AT1G70700 , AT3G57230 , AT5G46450 , AT3G23010 , AT2G32680 , AT1G18910 , AT2G24130 , AT3G59060 , AT1G61550 , AT5G59920 , AT5G12370 , AT1G25390 , AT4G22540 , AT1G25490 , AT1G04120 , AT1G56540 , AT5G18360 , AT2G14080 , AT5G11250 , AT4G05420 , AT2G33050 , AT5G13220 , AT4G03470 , AT1G78000 , AT5G51810 , AT5G41550 , AT3G46530 , AT2G33060 , AT4G03390 , AT5G38340 , AT2G27060
GO:0007165	signal transduction	32	1965	0.002	0.12	AT1G18890 , AT4G03460 , AT4G00710 , AT1G51480 , AT1G48090 , AT1G70700 , AT3G57230 , AT5G46450 , AT3G23010 , AT2G32680 , AT2G24130 , AT3G59060 , AT5G59920 , AT1G25390 , AT4G22540 , AT1G25490 , AT1G04120 , AT1G56540 , AT5G18360 , AT2G14080 , AT5G11250 , AT4G05420 , AT2G33050 , AT5G13220 , AT4G03470 , AT5G51810 , AT5G41550 , AT3G46530 , AT2G33060 , AT4G03390 , AT5G38340 , AT2G27060

Supp Table : Common gene names of the genes from GO:0006950 (response to stress) and GO:0050896 (response to stimulus)

geneid	gene_name
AT4G03510	RMA1
AT5G18100	CSD3
AT5G16960	AT5G16960
AT1G17880	BTF3
AT3G54050	HCEF1
AT5G65970	MLO10
AT1G71400	RLP12
AT2G33060	RLP27
AT3G03300	DCL2
AT4G03110	RBP-DR1
AT4G03550	GSL05
AT4G02260	RSH1
AT4G02580	AT4G02580
AT4G03470	AT4G03470
AT3G46940	DUT1
AT1G19350	BES1
AT4G11530	CRK34
AT1G19670	CLH1
AT4G37840	HKL3
AT4G00550	DGD2
AT1G61210	DWA3
AT5G14170	CHC1
AT4G23170	EP1
AT3G50980	XERO1
AT3G55990	ESK1
AT4G00830	LIF2
AT1G56540	AT1G56540

AT3G25560	NIK2
AT3G29320	PHS1
AT4G03260	MASP1
AT4G03460	AT4G03460
AT4G12010	DSC1
AT5G11630	NOXY2
AT1G01880	AT1G01880
AT1G19480	AT1G19480
AT1G01680	PUB54
AT4G08480	MAPKKK9
AT1G20020	FNR2
AT3G46530	RPP13
AT5G10140	FLC
AT1G12290	AT1G12290
AT2G24370	AT2G24370
AT5G58120	AT5G58120
AT4G02200	AT4G02200
AT1G61980	AT1G61980
AT4G23140	CRK6
AT2G34930	AT2G34930
AT1G59620	CW9
AT2G34810	AT2G34810
AT1G68530	KCS6
AT3G25510	AT3G25510
AT4G02280	SUS3
AT4G08500	MEKK1
AT2G43480	AT2G43480
AT5G11200	UAP56b
AT1G60930	RECQ4B
AT3G50950	ZAR1

AT1G69700	HVA22C
AT4G19230	CYP707A1
AT4G11470	CRK31
AT3G09710	IQD1
AT3G56700	FAR6
AT3G03050	CSLD3
AT3G07770	Hsp89.1
AT3G03250	UGP1
AT5G22330	RIN1/TIP49A
AT5G23540	AT5G23540
AT1G35720	ANNAT1
AT5G22290	NAC089
AT1G19730	ATTRX4
AT2G33050	RLP26
AT4G03280	PETC
AT2G21800	EME1A
AT4G02070	MSH6
AT4G02195	SYP42
AT2G14080	AT2G14080
AT4G11460	CRK30
AT2G17270	PHT3;3
AT5G11530	EMF1
AT4G22305	SOBER1
AT4G38360	LAZ1
AT5G11250	BURNOUT1
AT1G20160	ATSBT5.2
AT5G06780	EML2
AT1G01620	PIP1C
AT1G58390	AT1G58390
