# Supplementary material for: Multiple Sources of Introduction of North American Arabidopsis thaliana from across Eurasia
Source: Mol Biol Evol. 2021 Sep 9;38(12):5328–44. doi: 10.1093/molbev/msab268 (PMC8662644; doi:10.1093/molbev/msab268)
Supplement: msab268_Supplementary_Data [file msab268_supplementary_data.zip › Table S13.pdf]

Table S13. Collection years of the accessions used in the Bayesian phylogenetic analysis with BEAST

Hpg1		SouthIndiana4	
**Accession**	**Collection Year**	**Accession**	**Collection Year**
14INRCTx1	2014	G1_15INRC	2016
15INRCT26	2015	JK2524	1952
JK2511	1934	JK356	1897
JK2512	1956	801	1999
JK2513	1911	15INRCT42	2015
JK2514	1969	16INRCT00	2016
JK2516	1980	15INRCT17	2015
JK2518	1993	16INRCT07	2016
JK2527	1975	16INRCT06	2016
JK2530	1922		
JK346	1903		
JK376	1891		
JK389	1888		
JK395	1877		
JK399	1863		
S54	2015		
